# Role of Saponins from *Platycodon grandiflorum* in Alzheimer’s Disease: DFT, Molecular Docking, and Simulation Studies in Key Enzymes

**DOI:** 10.3390/molecules30081812

**Published:** 2025-04-17

**Authors:** Ashaimaa Y. Moussa, Abdulah R. Alanzi, Jinhai Luo, Jingwen Wang, Wai San Cheang, Baojun Xu

**Affiliations:** 1 Department of Pharmacognosy, Faculty of Pharmacy, Ain Shams University, Abbassia, Cairo 11566, Egypt; ashaimaa_yehia@pharma.asu.edu.eg; 2 Department of Pharmacognosy, College of Pharmacy, King Saud University, Riyadh 11451, Saudi Arabia; aralonazi@ksu.edu.sa; 3 Food Science and Technology Program, Department of Life Sciences, Beijing Normal-Hong Kong Baptist University, Zhuhai 519087, China; 4 State Key Laboratory of Quality Research in Chinese Medicine, Institute of Chinese Medical Sciences, University of Macau, Macao SAR, China

**Keywords:** Alzheimer’s, density functional theory, molecular dynamics simulations, saponins, Platycodon

## Abstract

Alzheimer’s disease (AD), one of the neurodegenerative disorders, afflicts negatively across the whole world. Due to its complex etiology, no available treatments are disease-altering. This study aimed to explore isolated saponins profiles from *Platycodon grandiflorum* in the binding pockets of six target proteins of AD using computational and quantum chemistry simulations. Initially, saponin compounds were docked to AD enzymes, such as GSK-3β and synapsin I, II, and III. The subsequent research from MD simulations of the best three docked compounds (polygalacin D2, polygalacin D, and platycodin D) suggested that their profiles match with the binding of standard active drugs like ifenprodil and donepezil to the six enzymes. Moreover, analyzing DFT quantum calculations of top-scoring compounds fully unravels their electronic and quantum properties and potential in anti-AD. The subtle differences between polygalacin D and D2, and platycodin D, were studied at the level of theory DFT/B3LYP, showing that the electron-donating effect of the hydroxy ethyl group in platycodin D rendering this compound of moderate electrophilicity and reactivity. Polygalacin D2 diglucoside substituent in position-2 contributed to its best binding and intermolecular interactions more than polygalacin D and prosapogenin D, which acted as the negative decoy drug.

## 1. Introduction

Neurodegenerative disorders are more than ever before afflicting populations worldwide. Around one billion people suffer from neurological and age-related ailments [1]; particularly, Alzheimer’s disease (AD), which is considered the fifth cause of death globally according to recent WHO reports [2]. By 2030, the number of AD American patients is prospected to rise by more than 35%, and the total AD cases may reach 150 million worldwide in 2050 [3].

The key symptoms of AD patients are cognitive and memory impairment, loss of self-care capacity, apathy, and agitation [4]. The pathological basis of these disorders is complicated and, so far, not totally understood, yet tau aggregation and phosphorylation as well as activation of the amyloidogenic pathway and oxidative stress seemed to be implicated in the progression of AD. The aforementioned disease pathologies pointed out the possible involvement of enzymes like NMDA, GSK-3β, BACE-1, AChE, BuChE, MAO-A, MOA-B, and ROCK2, whose alteration might occur concomitantly or separately [5,6]. In the meantime, effective treatments are missing that can completely cure AD, and even drugs that could partially manage the symptoms are limited to two classes only [7,8]. With the limited efficacy of previously discovered drugs like memantine, donepezil, galantamine, and rivastigmine, and their side effects, few options are left for patients. Currently, only aducanumab is used, which was introduced into the market in 2003 [9].

Based on several clinical studies, the multitargeted approach was proved to be the best choice for management of AD due to the complex nature of the disease and the need to induce or inhibit multiple genes and proteins. Patients who suffer from AD mainly have pathological hallmarks like neurofibrillary tangles and amyloid plaques, which are caused by the NMDA receptor (NMDA), BACE1, and GSK-3β hyperactivity. While β-secretase acts to cleave the β-amyloid protein to amyloid beta peptides 42 (Aβ-42) and subsequently to amyloid plaques, the excessive oxygen species can cause tau phosphorylation and neurofibrillary tangle formation. Moreover, the NMDA overactivity with the accumulation of amyloid plaques acts to develop neuronal death and toxicity [10]. In this regard, in silico experiments play an invaluable role; especially, with the current advancement of high-end computer power devices and the development of chemoinformatic methods to screen and optimize a large number of drug molecules in a time- and cost-effective manner [11,12,13,14].

### Saponins Against Age-Related Neurological Disorders

Why are natural products or functional foods preferred to treat neurodegenerative diseases? Rather than synthetic drugs, medicine foods are much favored; particularly, when a protective effect is required over a long period of time. Almost all humans need protective measures against neurodegenerative ailments. Plants have always been the repository of powerful secondary metabolites including alkaloids, phenolics, steroids, and saponins [15,16]. Saponins are bioactive natural products with a characteristic triterpene or steroidal aglycone core, and their effectiveness was long recognized in traditional Chinese medicine (TCM) [17]. Sun et al. referred to 33 saponins possessing a neuroprotective activity, either acting as antioxidants, inhibiting tau phosphorylation or ACHe inhibitors, or enhancing neuronal reconstruction and modulation of the GABA or NMDA receptors [17]. Ginseng was locally used in China to treat symptoms of AD [18], and its saponins effect on neuronal activity was recognized in several studies. A mixture of *Panax notoginseng* saponins composed of 14.5% notoginsenoside R1, 27.7% ginsenoside Rb1, and 28.0% ginsenoside Rg1 improved mitophagy and reduced cerebral oxidative stress in mice [19]. Ginsenoside Rg1 thwarted neuronal senescence via reducing NOX2-mediated ROS generation in H_2_O_2_-treated hippocampal neurons [20]. While short-term use of ginsenoside Rg1 reduced the level of amyloid β peptide with a notable memory improvement in OVX rats [21], their long-term use ameliorated redox stress and enhanced the plasticity proteins and activated progenitor cells in the hippocampus [22,23]. Moreover, ginsenoside Rg3 decreased amyloid β proteins 40 and 42 via a 2.9-fold increase in neprilysin gene expression [18]. Despite their violation of ADMET criteria, ginsenoside Rg3 proved to be effective in liposomal drug delivery systems instead of cholesterol to suppress the brain tumors in C6 glioma cells [24,25]. Furthermore, the drug delivery through an intranasal route together with β-cyclodextrin inclusion was efficient more than 150-fold to raise the brain concentration of gensinoside F1 [25]. Saponins of *P. grandiflorum* mainly comprised oleanolic acid with several sugar units attached; while platycodin (PD) possessed five sugars and PD3 contained six sugars, which contributed to the difference in their NO production or TNF-α activity. According to Wang et al., the less sugar units provided more potent pharmacological actions [26].

**Figure 1:**
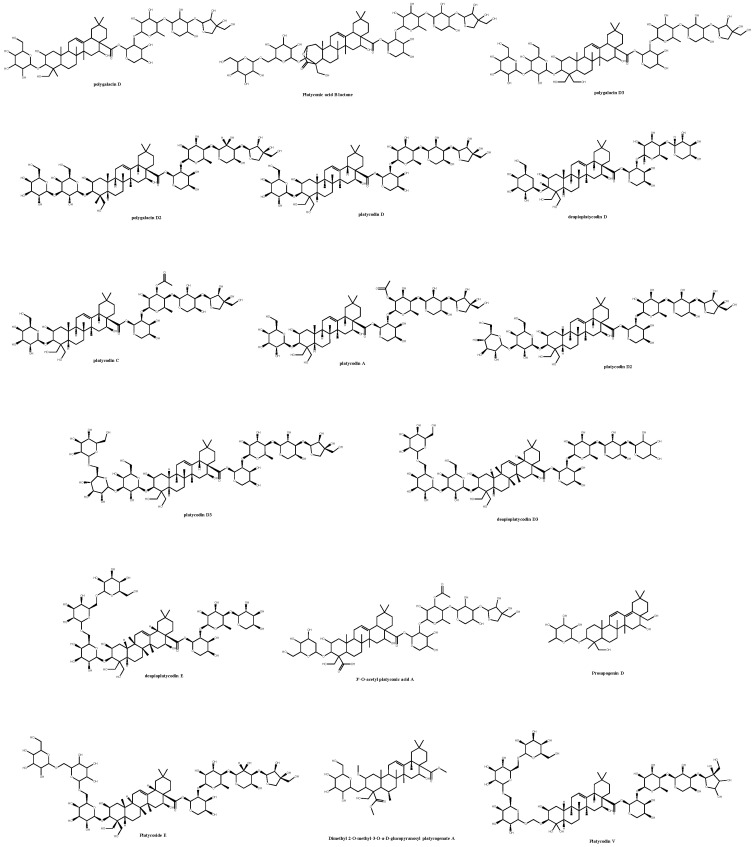
Chemical library of platycodin saponins subjected to computational analysis.

In this study, molecular docking and quantum chemistry simulations play an important role in predicting the binding capacity and interactions of *P. grandiflorum* saponins in the binding sites of key proteins involved in the neurodegenerative pathways. ADMET analysis was employed to indicate the oral bioavailability of these saponins and their nutraceutical use; dimethyl 2-O-methyl-3-O-a-D-glucopyranosyl, platycogenate A, polygalacin D2, platycodin C, platycodin D, prosapogenin D, deapioplatycodin D, platycodin D2, platycodin D3, deapioplatycodin D3, deapioplatycoside E, platyconic acid B lactone, platycodin A, 3″-O-acetylplatyconic acid A, polygalacin D, polygalacin D3, platycodin V, platycoside E, and polygalacin XI comprised the investigated list in this article (Figure 1).

## 2. Results

### 2.1. Selection of the Best Inhibitors for Proteins

The structures of the compounds were acquired from our previous work [24] based on their physicochemical properties. The top 15 compounds were selected for each protein target, synapsin I, synapsin II, synapsin III, BACE1, GSK-3β, and NMDA receptor. The selected compounds underwent a pharmacokinetic analysis. Table 1 presents the inhibitors chosen for the targeted proteins.

### 2.2. ADMET Results

All compounds were subjected to pharmacokinetics and ADMET criteria determination [27] where most of them showed poor BBB penetration and oral bioavailability, yet improvements in drug delivery systems might present a promising solution to enhance their therapeutic potential as was reported for ginsenosides.

### 2.3. Molecular Docking

Maestro 2018 conducted protein–ligand docking, which was further evaluated through MD simulation, RMSD, RMSF, radius of gyration, and the number of hydrogen bonds in protein–ligand interactions. A model system was formulated for polygalacin D2 as an inhibitor for synapsin I and BACE1 and platycodin D as the NMDA inhibitor. Polygalacin D was employed as an inhibitor of synapsin II, III, and GSK-3β. The type of interaction between the residues of the ligand–protein complex is shown in Table 2.

### 2.4. Molecular Quantum Calculations

Elucidating the quantum chemical characteristics of drug molecules is essential more than ever before to predefine interactions and reactivities especially before chemical synthesis [28]. The four compounds of platycodin D, polygalacin D, polygalacin D2, and prosapogenin D were selected to conduct the DFT calculations via the 6–31G (dp) basis set to reveal their key structural and electronic features (Table 3).

Electronegativity (χ) was determined as (I + A)/2 and the chemical potential (μ) was obtained as −(I + A)/2. Moreover, the global hardness (η) was attained as (I − A)/2, and the global softness was I/η. While ionization potential (I) was computed as −E_HOMO_, electron affinity (A) was the −E_LUMO_ value based on Koopman’s theorem.

$$\mu =\left(\frac{\partial E}{\partial N}\right)v(r)=-\left[\frac{IP+EA}{2}\right]=-\left[\frac{EHOMO+ELUMO}{2}\right]$$

ω or the electrophilicity index was realized from this equation ω = χ2/(2η), which describes the tendency of a molecule to accept electrons. Compounds chemical stability and reactivity were deduced from their energy band gap (ΔE) from the HOMO to the LUMO [29].

Polygalacin D showed a higher ΔE of 5.5984732 eV compared to prosapogenin D whose ΔE scored 0.18826 eV; thus, the former was of higher stability and lower chemical reactivity than the latter. Polygalacin D demonstrated a higher kinetic stability and lower reactivity with adequate hardness. Thus, it manifested a greater tendency to donate electrons with an EHOMO = −6.7048887 eV. On the other hand, the ELUMO was of lower energy 1.1064155 eV than −0.02371 eV, indicating its lower tendency to accept electrons. The ability of the selected compounds to acquire electron charges was noted from their electrophilicity index (ω), which was lower for polygalacin D than prosapogenin D. The electronegativity (χ) value indicating the affinity to attract electrons was 3.9056521 and 0.23538 for polygalacin D and prosapogenin D, respectively. The close values of global softness (σ) and hardness (η) in both compounds indicated their balanced electron density. Moreover, polygalacin D showed better charge separation signified by its high dipole moment (μ) of 7.8259 debye compared to prosapogenin D 4.8371 debye. Polygalacin D revealed its enhanced energetic stability based on its electronic energy value of (−17,794.6445474876) compared to prosapogenin D (−7977.93289844684). Polygalacin D2 and platycodin D were closely related to polygalacin D in most of their quantum chemical values; especially, their ΔE and electronic energy values of −25,782.40 and −22,130.38 Kcal/mol, respectively, denoting their stable energetic fitting, which matched their binding features in molecular docking and MD simulation results. Prosapogenin descriptors were not favored for good binding interactions inside the enzyme pockets.

### 2.5. Frontier Molecular Orbitals (FMOs)

**Figure 2:**
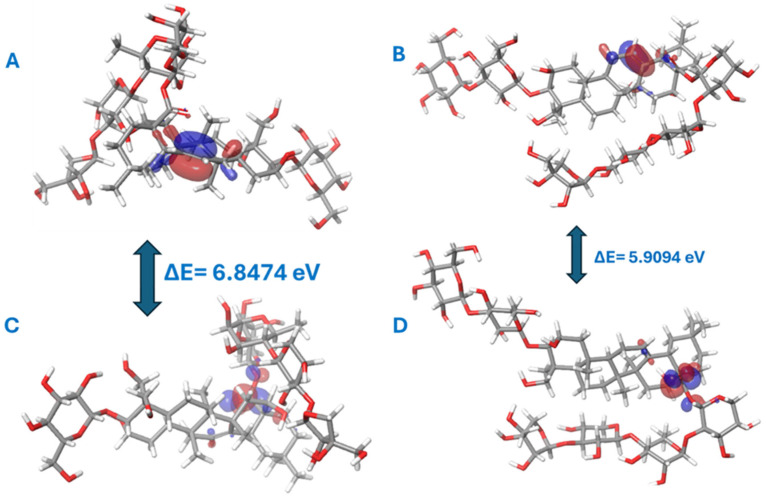
(**A**) Polygalacin D HOMO-LUMO, (**B**) prosapogenin D HOMO-LUMO, (**C**) platycodin D HOMO-LUMO, (**D**) polygalacin D2 HOMO-LUMO.

By analyzing the highest occupied molecular orbital (HOMO) and the least unoccupied molecular orbital (LUMO) of one of the promising compounds polygalacin D and prosapogenin D as a negative control, it was possible to infer the difference in their chemical stability and reactivity. This orbitals study promoted our understanding of a compound’s reactivity, electronic structure, and chemical interactions with other molecules. As far as HOMO is concerned, it controls the tendency to donate electrons in areas of lone pairs or bonding areas while the LUMO being vacant of electrons indicates the ability of the compound to accept electrons. The ΔE between the HOMO and LUMO is called the “energy band gap” and is given by the equation ΔE = |E_LUMO_ − E_HOMO_|. ΔE is a key indicator of the molecular reactivity during the quantum mechanical studies. Polygalacin D and D2 demonstrated its HOMO electron cloud throughout the C12 double bond. On the other hand, the LUMO featured a distribution over the C19-ester bond in half of the molecule. This arrangement manifested the possibility of electron donation from the HOMO in the central part of the molecule and the LUMO electron gain (Figure 2).

The three selected compounds varied in their 1- or 2-position substitutions in the pentacyclic aglycone core. After conducting docking analysis, this moiety was shown to fit in the same groove as the controls ifenoprodil and PF-04802, while the tetrasaccharide moiety might have rendered more stability via its prominent polar interactions outside the groove. The diglucoside in position 2 was favored as in polygalacin D2 in enzymes synapsin I, NMDA, and BACE1 followed by polygalacin D in synapsin II and III, which possessed one 2-glucose moiety with a single hydroxy ethyl group in position 1. These subtle disparities rendered different values of quantum chemical energies. Remarkably, polygalacin D2 showed the best interaction with all AD key enzymes, excluding GSK-3β, which indicated the importance of the second glucose unit in position 2 of the pentacyclic system. Platycodin D HOMO orbital was allocated to C11-C12 unsaturation, while the LUMO was detected in the C19-ester bond, and it possessed the highest ΔE energy gap of value 6.087462073 eV, denoting its stability.

In contrast, prosapogenin D HOMO orbitals were distributed over the double bonds C11-C12, C13-C18, and the C17 hydroxy methanol group single bond. The LUMO electron cloud was allocated over the single bond C12-C13 as well as atoms C11 and C17 in the attached methyl hydroxide. This electronic environment suggested that the HOMO orbital can donate electrons from the region of the conjugated double bonds C11-C12, C13-C18, and the LUMO can gain electrons in the region of the C12-C13 single bond and the C17 attached hydroxy methyl, which shed light over a high expected reactivity of this molecule (Figure 2).

The HOMO-LUMO gap was seen to be large in the three target compounds indicating their chemical stability and selectivity for the receptor properties.

From Table 3, Polygalacin D was the least prone to accept electrons due to its high LUMO orbital energy being the least nucleophilic. The HOMO-LUMO energy gap manifested that prosapogenin D is the most reactive and the softest compound with ΔE (0.1882 eV). Both polygalacin D2 and platycodin D were close in their ΔE values denoting favorable stability. The highest *χ* value was reported for polygalacin D, indicating its powerful ability to attract electrons compared to the non-docked compound prosapogenin D. The hardness index η was essential to indicate the compounds resistance to electronic distribution changes where polygalacin D was the hardest compared to the soft prosapogenin D (η = −0.0945). The Electrophilicity Index (ω) also matched with other descriptors pointing out polygalacin D2 as the most electrophilic candidate and the more prone to conduct intermolecular interactions with its high dipole moment of 8.0721 D and most negative electronic energy. On the other hand, platycodin D showed moderate stability with balanced nucleophilicity and electrophilicity.

### 2.6. Molecular Electrostatic Potential (MEP)

**Figure 3:**
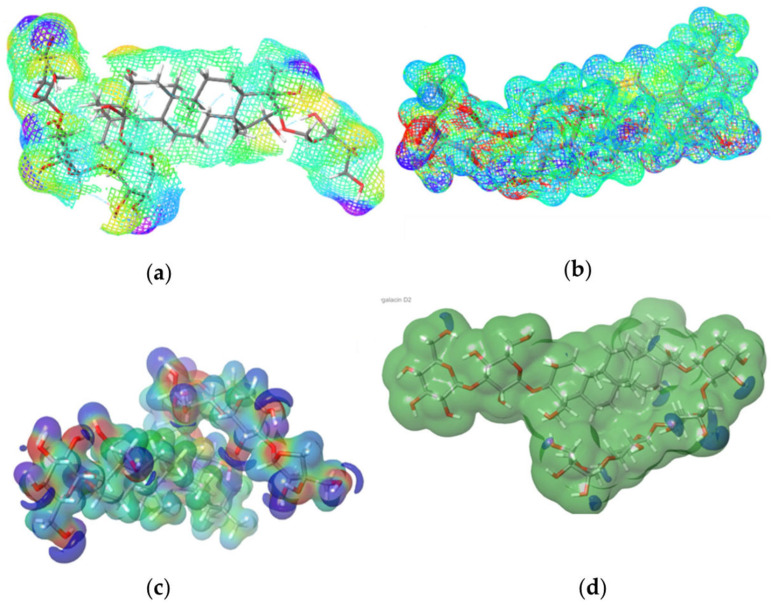
(**a**) Polygalacin D electrostatic potential, (**b**) prosapogenin D electrostatic potential, (**c**) platycodin D electrostatic potential, (**d**) polygalacin D2.

The MEP data of the three promising molecules were depicted compared to prosapogenin D as a negative control, being only docked by the NMDA protein, by employing DFT optimization with the B3LYP/6-311G (2d, p) functional and basis set (see Figure 3).

Platycodin D revealed multiple nucleophilic sites around the hydrogen atoms of the 2-glucoside moiety, likewise, around the hydroxy ethyl groups attached to position 1 of the aglycone. The electrophilic sites were noted around the heterocyclic oxygen of sugar. This part of the molecule is the same, which fitted in most of the bioactive sites in the enzymes investigated within the same docking location of the standards, which might refer to the potential significance of this 2-position attachment particularly with the planar conformation of the 2-glycoside rendering its insertion within the groove of interest feasible. The molecular electrostatic surface was displayed with color codes for polygalacin D, polygalacin D2, and prosapogenin D to unveil the active sites inside molecules. The green code manifested a neutral area. Whereas, the positive blue color indicated low electron density and regions favoring nucleophilic attacks, and the red color showed negative potential values and sites subjected to electrophilic attacks (Figure 3).

In both compounds, the nucleophilic regions were centered around oxygen atoms in the sugar rings or the oxygen substituents in C17 attached side chain, and the electrophilic sites were around the hydrogens attached to the hydroxyl groups. The rest of the molecule’s parts including the pentacyclic aglycone were mostly neutral. The MEP allows the prediction of hydrogen bonding and hence biological efficacy. According to the Mulliken-type charge distribution, the blue color denoted strong attraction around the alkyl groups and the red region indicated strong repulsion around the electronegative oxygen atoms, respectively. Consequently, polygalacin D and D2, and platycodin D, were suitable and compatible for AD enzyme binding.

### 2.7. Docking Interactions in Selected Proteins

#### 2.7.1. Synapsin I Receptor

**Figure 4:**
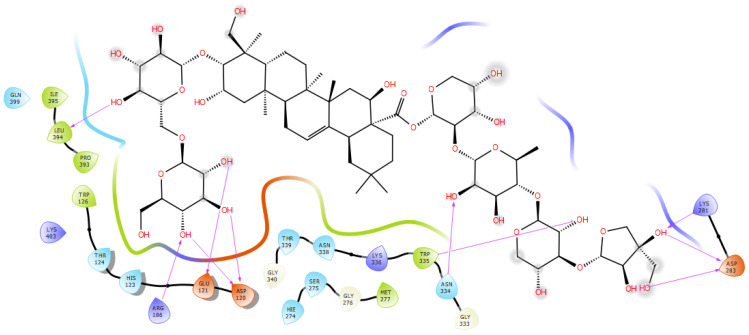
Polygalacin D2 in the binding site of synapsin I.

The saponins library was docked using AutoDock Vina software (v1.2.x) and ranked according to their binding free energies (ΔG) with the respective proteins. A standard molecule with reported anti-Alzheimer activity was used to compare the results as shown in Table 3 where the more negative values indicated a stronger and more stable binding interaction. All the investigated platycodin saponins exhibited a ΔG value below −4.67 Kcal/mol in synapsin I bind pocket with polygalacin D2 and 3″-O-acetyl platyconic acid scoring −6.74 and −6.36 Kcal/mol, respectively (Figure 4). These two compounds were elected to validate their binding interactions via molecular dynamics simulations. LY2811376, donepezil, and ifenoprodil were utilized as reference positive drugs. Polygalacin D2 superseded 3″-O-acetylplatyconic acid regarding the number of polar and nonpolar interactions (Table 2) [30].

**Figure 5:**
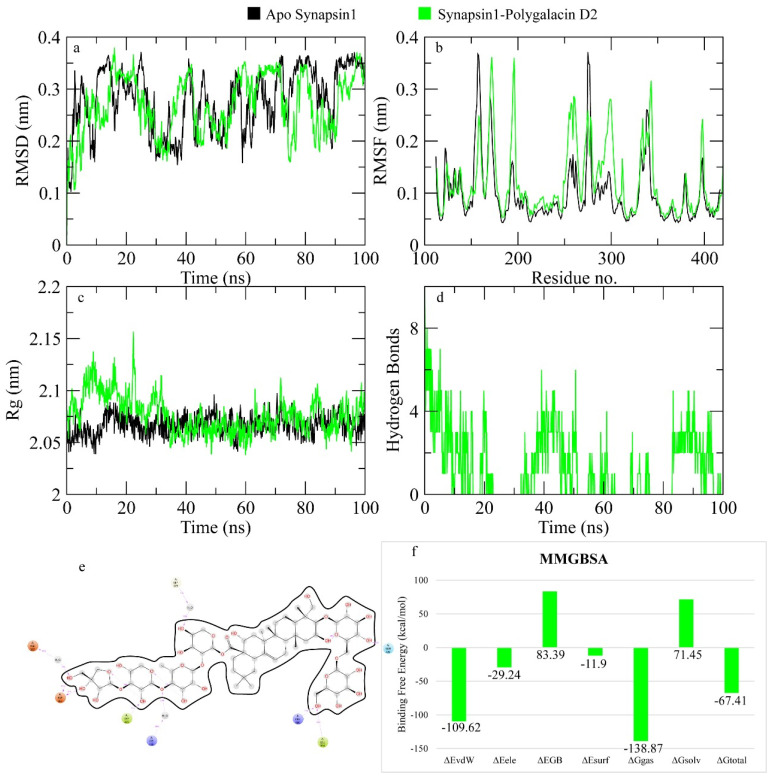
The simulation analysis. (**a**) The RMSD of Cα atoms of the apo protein and complex. (**b**) The flexibility of protein residues upon binding of the ligand. (**c**) Radius of gyration calculation of protein upon binding the ligand. (**d**) The hydrogen bonds calculation between the protein and ligand. (**e**) The molecular simulation result between polygalacin D2 and synapsin I. (**f**) The total binding free energy of energy components in the synapsin I-polygalacin D2 complex.

The RMSD of Cα atoms of Synapsin I bound to polygalacin D2 was measured and compared with the RMSD of the apo protein structure. The RMSD of the apo protein and complex deviated to 0.4 nm at 20 ns and then showed deviations in this range until the end, indicating the similar behavior in protein upon binding of the ligand (Figure 5). The RMSF plots revealed that the RMSF values of both the apo protein and complex were similar to most residues that exhibited values less than 0.2 nm (see Figure 5). Figure 5 shows the behavior of Rg during simulation. The plot revealed that the Rg values of the apo protein and complex were similar until 30 ns and then the Rg values of the complex showed deviations of 0.10 nm until the end of simulation. Figure 5 depicts the hydrogen bonds exhibited by the complex during the simulation run. At the beginning of the simulation, the protein–ligand complex formed six hydrogen bonds, which decreased to zero at some frames. The average number of hydrogen bonds was three during the simulation. The total binding free energy of the complex was −67.41 ± 1.64 Kcal/mol. The contribution of other energy parameters is shown in Figure 5**.**

Polygalactia D2 revealed around 10 hydrogen bonds to several amino acid residues as Asn 334, Trp335, and Lys281 in a manner that resembles the standard drugs LY281137 and donepezil: additionally, nonpolar interactions with the hydrophobic bed formed by ile395, Pro393, Trp126, Met277, Gly276, Met297, and Ser275. His123, Thr124, and Asn338 explained its favorable binding free energy −6.742 Kcal/mol that surpassed the controls (see Figure 4). Different descriptors such as RMSD, RMSF, and Rg were measured and all indicated the stability of ligands binding. The Rg values indicated the compactness of the protein after binding to polygalacin D2 and 3″-O-acetyl platyconic acid (Figure 5).

#### 2.7.2. Synapsin II Receptor

**Figure 6:**
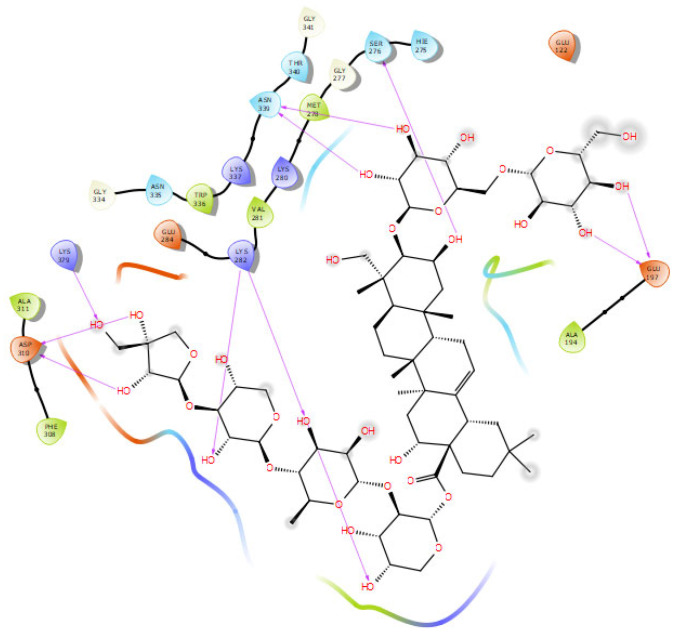
Docking interactions of polygalacin D2 in the binding site of synapsin II.

The results of docking, MD simulations, and MMGBSA of synapsin II receptor with polygalacin D, polygalacin D2, and donepezil (control) are shown below. As shown in Table 4, the binding free energies for the docked molecules ranged between −3.712 and −6.101 Kcal/mol. Polygalacin D and polygalacin D2 were the candidates selected for further molecular dynamics measurements since they scored the lowest energy values −6.011 and −6.101 Kcal/mol, respectively. The docked complex of synapsin II receptor–polygalacin D2 is shown in Figure 6.

Polygalacin D2 formed polar interactions with residues asp310, lys379, lys282, asn339, ser276, and glu197 via 11 hydrogen bonds that ranged in their length between 1.65 and 2.50 A. Despite the variation in molecular size, polygalacin D2 effectively fitted in the 1i7L binding site as standards PF-0480, LY-2811376, and ifenoprodil with amino acid contacts comprising phe308, trp336, lys280, ala311, gly277, and lys337, which formed both van der Waals and hydrophobic interactions.

**Figure 7:**
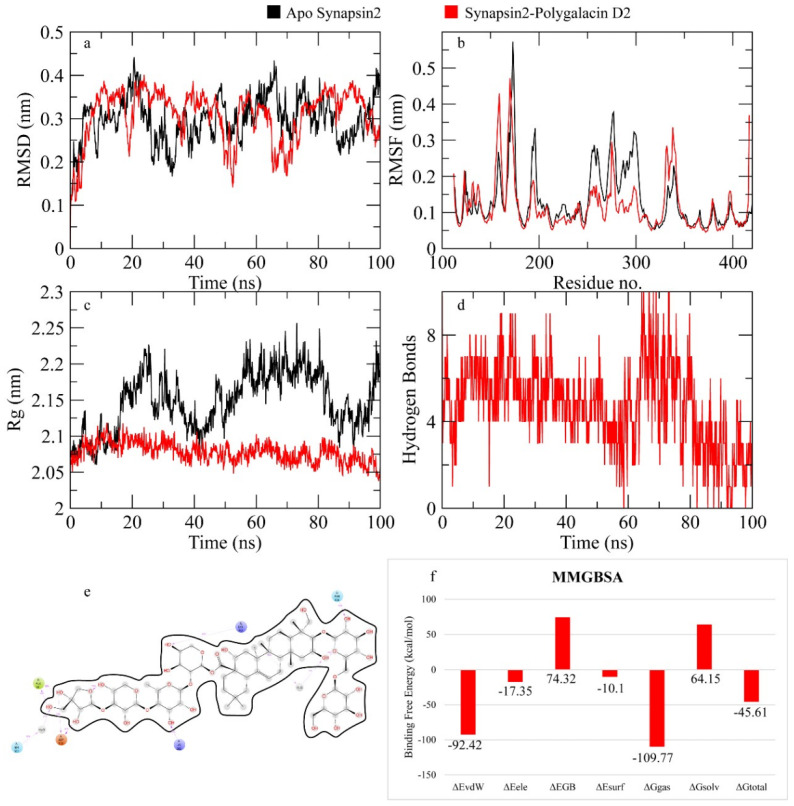
The simulation analysis. (**a**) The RMSD of Cα atoms of the apo protein and complex. (**b**) The flexibility of protein residues upon binding of the ligand. (**c**) Radius of gyration calculation of protein upon binding the ligand. (**d**) The hydrogen bonds calculation between the protein and ligand. (**e**) The molecular simulation result between polygalacin D and synapsin II. (**f**) The total binding free energy of energy components in the synapsin II–polygalacin D complex.

The RMSD of Cα atoms of synapsin II bound to polygalacin D was measured and compared with the RMSD of the apo protein structure. The RMSD of the apo protein and complex deviated to 0.4 nm at 20 ns and then showed deviations in this range until the end, indicating the similar behavior in protein upon binding of the ligand (Figure 7). The RMSF plots revealed that the RMSF values of both the apo protein and complex were similar as most residues exhibited values less than 0.2 nm. Figure 7 shows the behavior of Rg during simulation. The plot revealed that the Rg values of the apo protein and complex were similar until 20 ns and then the Rg values of the apo protein showed deviations of 0.15 nm until the end of the simulation. Figure 7 depicts the hydrogen bonds exhibited by the complex during the simulation run. At the beginning of the simulation, the protein–ligand complex formed six hydrogen bonds, which decreased to three at some frames. The average number of hydrogen bonds was four during the simulation. The total binding free energy of the complex was −45.61 ± 1.69 Kcal/mol. The contribution of other energy parameters is shown in Figure 7.

#### 2.7.3. Synapsin III Receptor

The docking results, MD simulations, and MMGBSA of synapsin III receptors with polygalacin D and polygalacin D2 compared to ifenoprodil, donepezil, and PF-0480 (control) are shown below.

**Figure 8:**
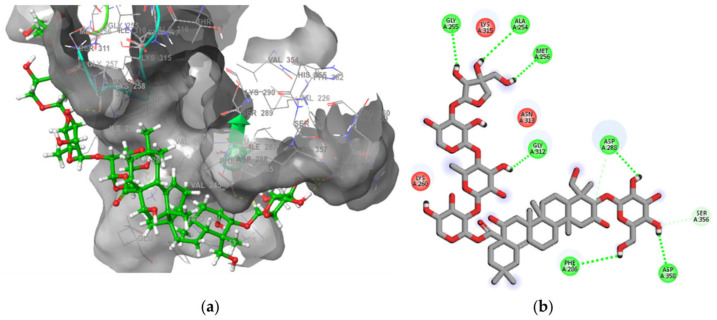
(**a**) The docked complex of synapsin III receptor–polygalacin D. (**b**) Docking interactions of polygalacin D in the binding site of synapsin III.

All the selected saponins were docked into the binding site of synapsin III and scored between −3.202 and −6.145 Kcal/mol. Based on the calculation of the binding free energies, the candidates polygalacin D and polygalacin D2 were employed for further molecular dynamics studies (see Table 4). The positive controls were known as CNS stimulants and dopamine reuptake inhibitors. Upon investigating the binding modes, we could infer that both molecules formed several hydrogen bond interactions and were accessible to the hydrophobic bed of the enzymatic pocket but in a different way from PF-04802367, which reflects the different orientation inside the receptor groove (see Table 2, Figure 8). Common residues between the control and the two binding ligands were phe286, trp314, ala254, and ile287 (Figure 8). Both docked compounds fitted horizontally without entering the narrow hole characteristic for the controls, yet they revealed better stability and enhanced binding features, which might be justified by the larger surface area and greater number of hydrogen bonds as well as nonpolar interactions (see Figure 8).

**Figure 9:**
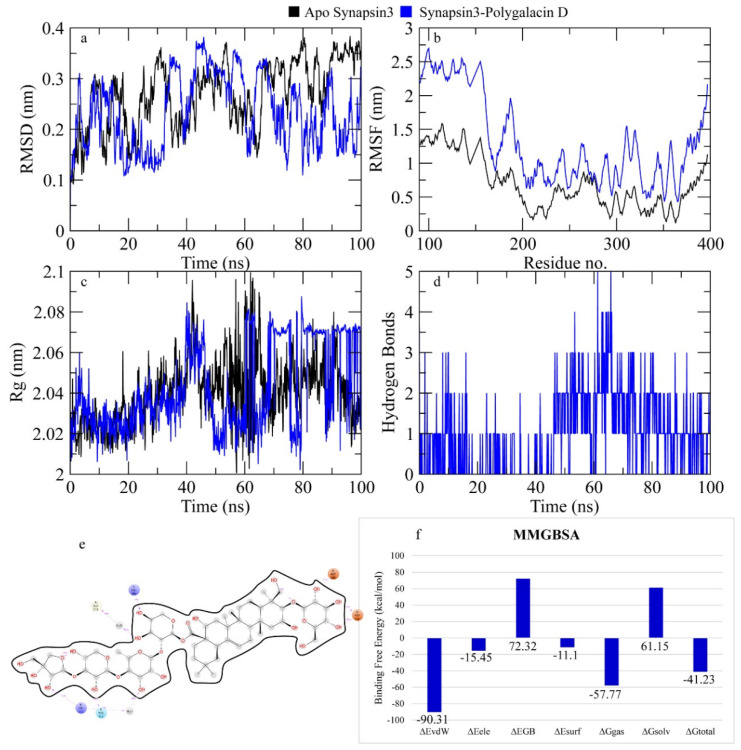
The simulation analysis. (**a**) The RMSD of Cα atoms of the apo protein and complex. (**b**) The flexibility of protein residues upon binding of the ligand. (**c**) Radius of gyration calculation of protein upon binding the ligand. (**d**) The hydrogen bonds calculation between the protein and ligand. (**e**) The molecular simulation result between polygalacin D and synapsin III. (**f**) The total binding free energy of energy components in the synapsin III–polygalacin D complex.

The RMSD of Cα atoms of Synapsin III bound to polygalacin D was measured and compared with the RMSD of the apo protein structure. The RMSD of the apo protein and complex deviated to 0.3 nm at 20 ns and then showed deviations in the range of 0.3–0.4 nm until the end, indicating the similar behavior in protein upon binding of the ligand (Figure 9). The RMSF plots revealed that the RMSF values of both apo proteins were less than the complex, where the RMSF of N terminal reached 2.5 nm while the RMSF values of N terminal in the apo protein were around 1.5 nm. Figure 9 shows the behavior of Rg during simulation. The plot revealed that the Rg values of the apo protein and complex were similar until 40 ns and then the Rg values of the apo protein and complex showed deviations of 0.06 nm in the second half of the simulation. Figure 9 depicts the hydrogen bonds exhibited by the complex during the simulation run. At the beginning of the simulation, the protein–ligand complex formed three hydrogen bonds, which decreased to one at some frames. The average number of hydrogen bonds was two during the simulation. The total binding free energy of the complex was −41.23 ± 0.05 Kcal/mol. The contribution of other energy parameters is shown in Figure 9.

#### 2.7.4. N-Methyl-D-Aspartate (NMDA) Receptor

The results of docking, MD simulations, and MMGBSA of the NMDA receptor with polygalacin D and ifenprodil (control) are shown below.

The NMDA receptor is comprised of two subunits: the GluN1 and the GluN2 assembling as a heterodimer. While GluN1 binds glycine or D-serine as a co-agonist, GluN2 binds glutamate as an agonist [31]. The domains of each subunit can be categorized into four types: the ligand-binding domain (LBD), the amino-terminal domain (ATD), the cytoplasmic C-terminal domain, and the transmembrane domain (TMD) [32].

**Figure 10:**
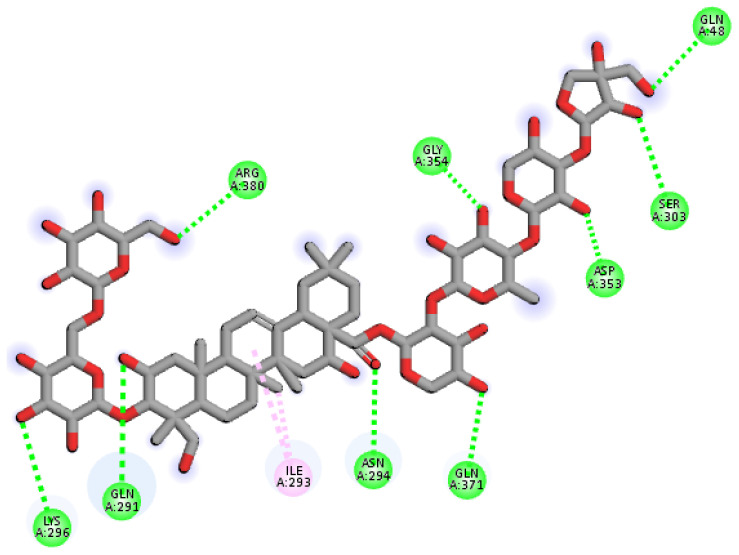
Polygalacin D2 in the binding site of the NMDA receptor.

The docked complexes and residue-specific interactions of the NMDA receptors with polygalacin D2, polygalacin D, and donepezil (control) are illustrated in Table 2, respectively. Polygalacin D2 and platycodin D were docked in a similar lateral orientation featuring a new location distinct from the standards, yet displayed better glide docking scores of −7.755 and −7.296 Kcal/mol, respectively. Polygalacin D2 manifested hydrogen bonds to the key residue lys296 with distance 2.51 A and gln291 with distances 2.31 and 2.34 A (Figure 10) and extended hydrophobic forces to leu280, leu377, met375, ile275, and leu292 as well as ile293, val355, and ala307, which are characteristic to the binding features of ifenoprodil and ly-2811375.

**Figure 11:**
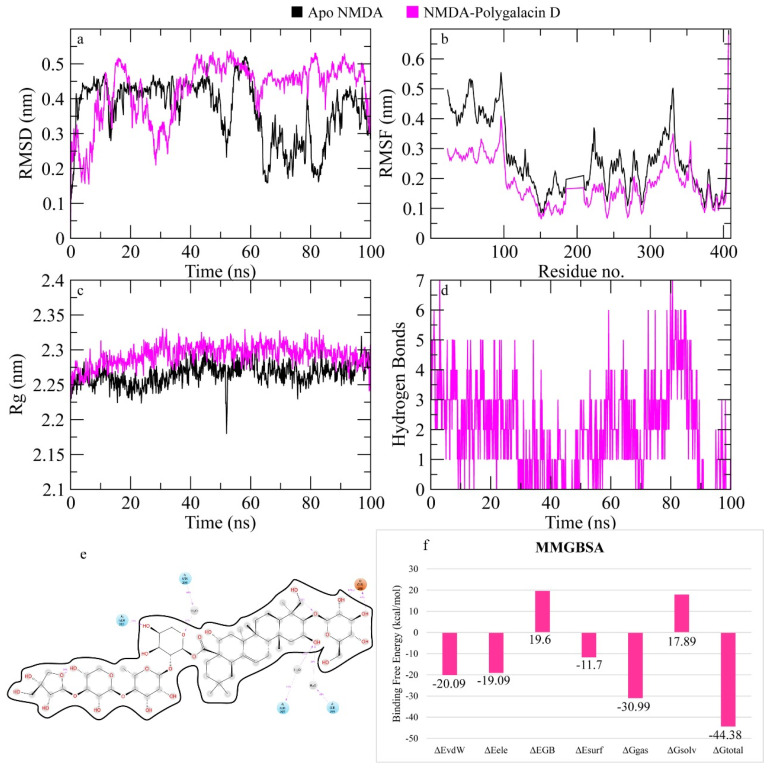
The simulation analysis. (**a**) The RMSD of Cα atoms of the apo protein and complex. (**b**) The flexibility of protein residues upon binding of the ligand. (**c**) Radius of gyration calculation of protein upon binding the ligand. (**d**) The hydrogen bonds calculation between the protein and ligand. (**e**) The molecular simulation result between polygalacin D and NMDA. (**f**) The total binding free energy of energy components in the NMDA–polygalacin D complex.

The RMSD of Cα atoms of NMDA bound to polygalacin D was measured and compared with the RMSD of the apo protein structure. The RMSD of the apo protein deviated to 0.45 nm at 10 ns and then showed a decline in values of 0.2 nm during the 60 to 80 ns interval. After 80 ns, it showed deviations in the range of 0.4 nm, while the RMSD of the complex was comparable to the apo protein but it was more than the apo protein in the last part of the simulation (Figure 11). The RMSF plots revealed that the apo protein showed more fluctuations than the complex as the complex values remained less than 0.2 nm while apo protein values were around 0.3 nm. Figure 11 shows the behavior of Rg during simulation. The plot revealed that the Rg values of both the apo protein and complex demonstrated stability throughout the simulation beginning from ~2.25 nm and consistently ranged from ~2.25 to 2.3 nm throughout the simulation run, indicating the stability of the protein structure upon binding of the ligand. Figure 11 depicts the hydrogen bonds exhibited by the complex during the simulation run. At the beginning of the simulation, the protein–ligand complex formed seven hydrogen bonds, which decreased to two at some frames. The average number of hydrogen bonds was three during the simulation. The total binding free energy of the complex was −44.38 ± 1.23 Kcal/mol. The contribution of other energy parameters is shown in Figure 11.

#### 2.7.5. GSK-3β

Inhibition of GSK-3β might improve aging brain symptoms and reduce memory deficits and detrimental AD events; especially, the neuronal death induced by amyloid β and tau hyperphosphorylation. GSK-3β with its matching oligonucleotide structure as tau protein kinase I plays a focal role in forming the tau helical filaments that develop into neurofibrillary tangles disrupting brain function [33,34].

**Figure 12:**
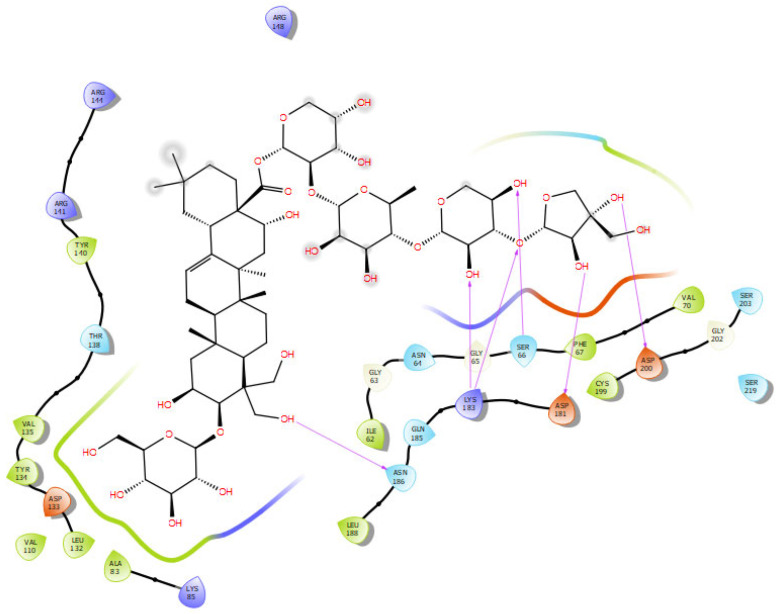
Docking interactions of platycodin D in the binding pocket of GSK-3β.

The best binding scores were reserved for platycodin D, which successfully inserted its pentacyclic ring into the nonpolar enzymatic groove like the controls used (Figure 12). The attached sugar residues extended to form many of the polar contacts outside this hole with a binding score of −7.147 Kcal/mol compared to PF-04820. It seemed that this hydrophobic groove represented a crucial bioactivity requirement since all the controls satisfied this condition. Moreover, combining both internal and external interactions exhibited the best-fitting scores and was only effectively achieved by platycodin D. It is worth noting that prosapogenin D bound inside the hydrophobic hole but without a polar external side chain and polygalacin D as well as platycodin D3 passed around the hydrophobic groove favoring the more polar hydrogen bond interactions outside.

The platycodin D sugar part formed stable polar bonds with asp200, asp181, lys183, ser66, and asn186; whereas, its aglycone interacted with val70, phe67, cys199, ile62, leu188, ala83, leu132, val110, tyr134, val135, and tyr140, where most of them matched the used standard drugs.

**Figure 13:**
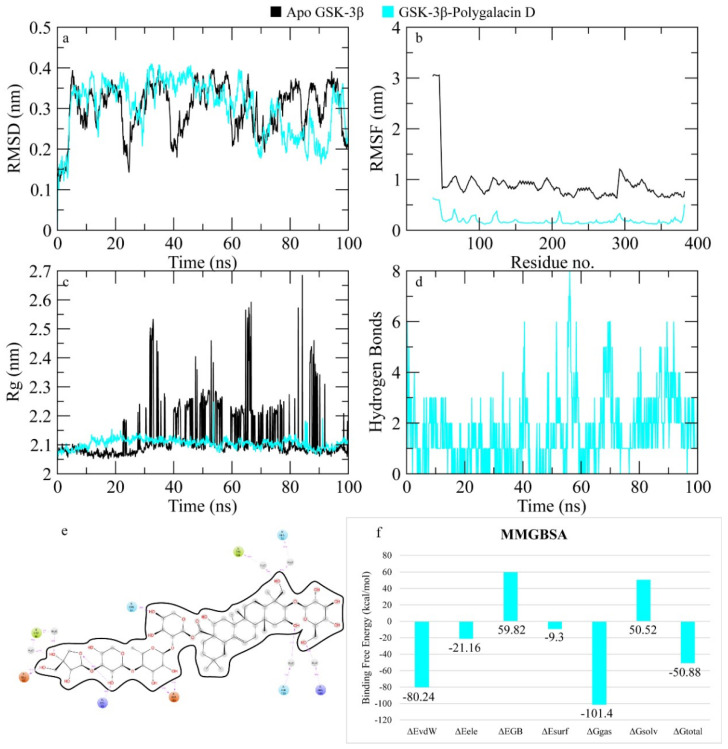
MD simulation measurements of the GSK-3β–polygalacin D complex. (**a**) The RMSD of Cα atoms of the apo protein and complex. (**b**) The flexibility of protein residues upon binding of the ligand. (**c**) Radius of gyration calculation of protein upon binding the ligand. (**d**) The hydrogen bonds calculation between the protein and ligand. (**e**) The molecular simulation result between polygalacin D and GSK-3β. (**f**) The total binding free energy of energy components in the GSK-3β–polygalacin D complex.

The RMSD of Cα atoms of GSK-3β bound to polygalacin D was measured and compared with the RMSD of the apo protein structure to find the complex stability during simulation. The RMSD of the apo protein deviated to 0.4 nm at 10 ns and then showed a decline in value of 0.2 nm during the 10 to 20 ns intervals. After 20 ns, it showed deviations in the range of 0.2–0.4 nm throughout the time, while the RMSD of complex was comparable to the apo protein but it was lower than the apo protein in the last part of the simulation (Figure 13). The RMSF plots revealed that the apo protein showed more fluctuations than the complex as the complex values remained less than 0.5 nm while the apo protein values were around 1 nm (Figure 13). Figure 3c shows the behavior of Rg during simulation. The plot revealed that the Rg values of the apo protein showed deviations throughout simulation while the Rg values of the complex demonstrated stability throughout the simulation beginning from ~2.0 nm and consistently ranged from ~2.1 to 2.2 nm throughout the simulation run, indicating the stability of the protein structure upon binding of the ligand. Figure 13 depicts the hydrogen bonds exhibited by the complex during the simulation run. At the beginning of the simulation, the protein–ligand complex formed six hydrogen bonds, which decreased to one at some frames. The average number of hydrogen bonds was three during the simulation. Moreover, the binding free energy of the complex was calculated by employing the MMGBSA methods. It is calculated by extracting the sum of the receptor and ligand free energy from the total energy of the complex. Several energy components such as electrostatic contribution, van der Waals contribution, and solvation energy in a generalized Born environment were measured. The total binding free energy of the complex was −50.88 ± 1.07 Kcal/mol. The contribution of other energy parameters is shown in Figure 13.

#### 2.7.6. BACE1

**Figure 14:**
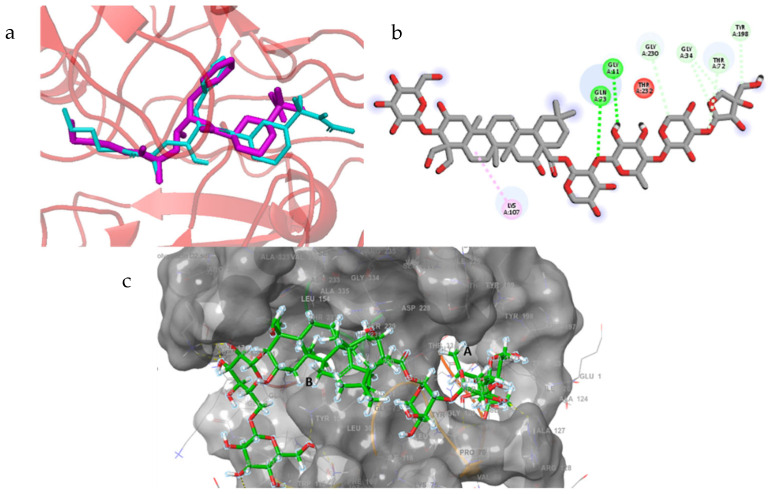
Redocking of the co-crystallized native ligand in the BACE1 enzyme. (**a**) Superimposition of the docked and co-crystallized native ligand (**b**) Docking interactions of platycodin D in the binding pocket of BACE1. (**c**) Polygalacin D2 in the binding pocket of BACE1.

The native co-crystallized ligand of the BACE1 receptor was redocked and illustrated in Figure 14 with an RMSD value of 0.23.

Polygalacin D2 scored the lowest binding free energy of −7.298 Kcal/mol on account of its larger molecule that spanned a wider surface area of the enzyme and interacted with tyr198, arg128, pro70, and ser36 in the same way as the controls as well as with additional hydrogen bonds to asn111, phe109, arg307, asp228, and lys107 (see Table 2) in a neighboring binding groove (Figure 14). Briefly, a larger number of polar interactions might explain the polygalacin D2 better fitting ability and leave it to be investigated via in vitro studies for its possible biological effects (Figure 14). Polygalacin D2 was the only molecule that displayed sufficient polar contacts matching those of the standards. Further analysis of the rest of the docked structures revealed their location far from groove A or even both grooves A and B; thus, lacking an adequate distance to interact appropriately with the enzyme.

**Figure 15:**
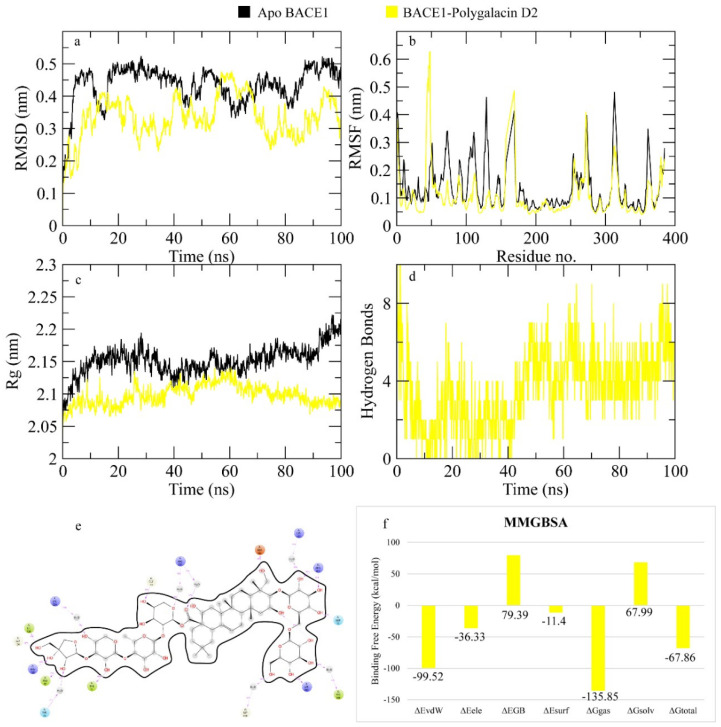
MD simulation data of the BACE1– polygalacin D complex. (**a**) The RMSD of Cα atoms of the apo protein and complex. (**b**) The flexibility of protein residues upon binding of the ligand. (**c**) Radius of gyration calculation of protein upon binding the ligand. (**d**) The hydrogen bonds calculation between the protein and ligand. (**e**) The molecular simulation result between polygalacin D and BACE1. (**f**) The total binding free energy of energy components in the BACE1–polygalacin D complex.

The RMSD of Cα atoms of BACE1 bound to polygalacin D was measured and compared with the RMSD of the apo protein structure. The RMSD of the apo protein deviated to 0.5 nm at 20 ns and then showed deviations in this range until the end, while the RMSD of the complex was lower than the apo protein (Figure 15). The RMSF plots revealed that the RMSF values of both the apo protein and complex were similar as most residues exhibited values less than 0.2 nm. Figure 15 shows the behavior of Rg during simulation. The plot revealed that the Rg values of the apo protein were more than the complex, but both demonstrated stability throughout the simulation. Figure 15 depicts the hydrogen bonds exhibited by the complex during the simulation run. At the beginning of the simulation, the protein–ligand complex formed five hydrogen bonds, which decreased to two at some frames. The average number of hydrogen bonds was three during the simulation. The total binding free energy of the complex was −67.86 ± 1.67 Kcal/mol. The contribution of other energy parameters is shown in Figure 15.

## 3. Discussion

*P. grandiflorum* is one of the famous medicine food homology plants in the *Campanulaceae* family, growing mainly in Korea, Japan, East Siberia, and China, whose potential in treating AD has not been investigated before despite its wide availability and popular use [35]. This highly nutritional popular appetizer comprises many beneficial compounds such as polyphenols, flavonoids, and saponins, which display activities such as hepatoprotective, antioxidant, hypolipidemic, anti-inflammatory, and anticancer effects [35]. Moreover, *P. grandiflorum* was previously described in Japan medicinal history and used as food and medicine during the long Chinese and East Asian traditional medicine records [36]. Whereas, the polar extracts of *P. grandiflorum* ameliorated amnesia and reversed memory impairment, few studies dealt with purified saponins and their activity against neuronal inflammation or amnesia [37], among which only platycodin D showed an antioxidant effect and neuroprotective role by elevating AMPK activation both in vivo and in vitro [38]. Moreover, 2″-*O*-acetyl-polygalacin D2 and platycodin D protected against ischemic reperfusion injury in the hippocampus [39]. In this study, we aimed to virtually analyze a list of 18 saponins isolated from *P. grandiflorum* by means of molecular docking, density functional theory, MD simulations, and MMGBSA energy calculations in the binding sites of several critical enzymes involved in AD pathology—the NMDA; BACE1; synapsin I, II, and III; and GSK-3B—consequently, paving the way to the development of multitarget molecules that can treat, ameliorate, or stop the progression of AD. This saponins list bears structural similarity to the ginsenosides of *Panax ginseng*, in that neuroprotective and antioxidant effects were proven both in vitro and in vivo [22].

With about 2% saponin content, the genus *Platycodon* is rich in oleanane triterpenoidal saponins comprised of C30 structures arranged in 4 or 5 ring configurations. The C3 and C28 formed ether and ester links, respectively. While various sugar types formed the hydrophilic side of the molecule, aglycones shaped the hydrophobic side. The attached sugars are mostly D-rhamnose, D-glucose, D-xylose, D-arabinose, and D-apiose [40]. This chemical skeleton largely matched ginseng saponins; particularly, ginsenoside Ro with its oleanolic acid core structure [26], which motivated the authors to embark on this study. De-glycosylated platycosides were more active than their glycosides due to their better bioavailability and BBB accessibility [41,42]. Yet, advancements in the field of drug delivery and inclusion opened the way for investigating the whole saponin molecules as well. C4 substituents such as methyl, carboxylic, or hydroxy methyl groups provided more diversity of the platycoside backbone forming up to 30 saponins including deapiplatycodin D3, deapiplatycoside E, platycoside E, platycodin A, platycodin D3, dPD, PD, polygalacin D, 2″-O-acetyl polygalacin D, and 3″-O-acetyl polygalacin D [43].

Synapsin I, II, and III interactions with the functional α-helical αSyn can aggravate AD symptoms and motor disabilities. Bioactive molecules as MPH act as synapsin silencing agents to destabilize fibril formation [44]. Many protein–ligand complexes in a variety of docked conformations were generated using AutoDock Vina V1.12 and AutoDock 4 as tools for computer-aided drug design to serve for the prediction of the receptor binding interactions. Selection of the final poses was conducted based on the free energy scores of ∆G values. Ligand molecules comprised of the 15 saponins list and positive controls were docked in the selected targets of BACE1, synapsin I, II, III, GSK-β, and NAMD to accurately select the poses with highest binding affinities, which can modulate the target proteins.

Polygalacin D was reported as an apoptotic and antitumor agent against hepatocellular carcinoma cells and the HSC-T6 cell line [45,46]. The mechanism was inferred to proceed via the PI3K/Akt pathway in non-small cell lung cancer [47]. Its anti-inflammatory effect was revealed by suppressing NF-kB activation [48]. In this study, polygalacin D manifested promising computational results that support its prospected neurodegenerative and anti-AD activity. Polygalacin D showed stable interactions with the six enzymatic targets. In the synapsin II binding pocket, PGD assumed a molecular orientation that largely matched donepezil with hydrophobic contact to Ser276 and hydrogen bonds to Asn339 (2.97 Å) and Asn339 (2.86 Å) residues. The ΔG value was among the best scores achieved in the whole saponin screened library −12.3 Kcal/mol. Molecular dynamics simulations showed a larger extent of persistent interactions such as Glu122, Glu148, Gln188, Asn339, and Arg187 with 2-hydroxyl glucosyl and 2-hydroxy apiosyl groups featuring the core interactions of donepezil in the same subunit.

The FMOs calculations also displayed a low ΔE value between the HOMO and LUMO, suggesting the stability of polygalacin D to interact with the enzymatic pocket residues. The MEP revealed a complementary electrostatic potential between the candidate molecule polygalacin D and many of the standard molecules.

Recently, the treatment direction of AD was shifted towards combination therapies of both amyloid- and tau protein–directed drugs, which is regarded as a breakthrough particularly in late stages when both proteins are abundant [21]. Although the accurate role of any of these proteins is still undiscovered, their implication in AD pathology is well confirmed. Therefore, double-targeting and applying preventive measures as well might represent a successful approach.

An intricate relation exists between synapsin I, BACE1, and NMDA, where synapsin I upregulates BACE1/β-secretase activity enhancing Aβ production. This commences via the α-secretase cleavage of APP releasing sAPPα, which is further modified by γ-secretase to generate the intracellular domain of APP [49]. BACE1 as an aspartate protease acts on the aforementioned domain to produce sAPPβ, the precursor of Aβ. The abnormal levels of Aβ bind with the voltage-gated Ca^2+^ channel NMDA receptor to block Ca^2+^ flow and express the etiology of AD [50]. BACE1 enzyme is an important target to ameliorate AD with its higher levels supporting higher Aβ production in the diseased brain compared to the normal one [51,52].

Upon activation, BACE1 displayed its four N-glycosylation sites and cysteine residues that manifested three disulfide bonds necessary for enzymatic activity. Moreover, the BACE1 conformation suitable for substrate binding was the flap-closed conformation, which was stabilized via substrate binding to a cleft in BACE1 with amino acids Thr72, Arg235, Ser328, and Thr329. These contacts were clearly noticed in polygalacin D justifying its binding energy of −5.607 Kcal/mol; yet, in polygalacin D2, further polar contacts were formed up to 15 hydrogen bonds (see Table 4) giving rise to a Gibbs free energy of −7.298 Kcal/mol. Fortunately, polygalacin D2 interacted with the same amino acids as the controls ifenprodil, PF-04820, and donepezil in the same groove and with a matching stable orientation. For instance, the 1-ethyl hydroxy group with glu265; the 2 and 3-OH of the first glucose unit in position 2 with lys321, gly264, and arg307; as well as the 3,4,6-OH of the second glucose unit in position 2 with asn111, phe109, lys107, and gln73. Further interactions as hydrogen bonds between the xylosyl unit with tyr198, and the apiosyl unit with arg128, ile126, pro70, and ser36 were only seen in polygalacin D2. Similar residue contacts were seen in rosmarinic acid, donezepil, and ursolic acid binding with BACE1; particularly, with gly235, Thr329, Arg235, and Phe113, respectively [53]. Mirza et al. pointed out good binding interactions of rosmarinic, ursolic, and carnosic with synapsins I, II, and III in a manner highly matching polygalacin D2 and close binding energy values [53].

Polygalacin D2 showed an anti-neuroinflammatory effect by reducing Th1 and Th2 cytokines in microglial cells and protected against reperfusion injury in the gerbil hippocampus [31,54]. Moreover, its activity was prominent in cancer research as a kinase inhibitor and apoptotic agent in Chinese hamster and lincomycin resistant cancer tissues [48]. Polygalacin D2 demonstrated promising binding to key proteins involved in the etiology of AD such as NMDA, BACE1, and synapsin III, II, and I, showing the lowest Gibbs free energy value of −7.755, −7.298, −5.882, −6.011, and −6.742 Kcal/mol, respectively.

Further confirmation was established by MD simulation analysis based on the RMSD and RMSF values. Polygalacin D2 possesses major functional groups as hydroxyl groups in positions 1, 3, and 16, as well as those in the glucose disaccharide interacting with lys107, asn111, phe109, arg307, and lys321, and the tetra saccharide units comprised of arabinose, mannose, xylose, and apiose interacting with tyr198, arg128, ile126, and ser36. Additionally, the 12,13-unsaturation in ring C was the contact point with several hydrophobic residues. These outcomes refer strongly towards the BACE1 modulating effect of polygalacin D2, which might contribute to the AD disease-altering activity of this molecule.

From all platycodon saponins, polygalacin D and D2 demonstrated the most favorable binding affinity towards synapsin proteins I, II, and III. Synapsins are involved in proper synaptic maturation and plasticity, and their loss of function or expression impairment is a necessary concern after AD development [49]. Herein, we introduced polygalacin D and D2 as two promising molecules with a combined promising effect in BACE1 and synapsin proteins in AD; yet, further studies in in vitro and in vivo models are demanded for a holistic approach. Despite its high computational costs, MD simulation results provided a more accurate drug design model through its flexible binding modules with the receptor and adding up the effect of surrounding water molecules.

NMDA represented one enzyme that is affected by high levels of tau pathology and is one of the alternative targets in the case of inefficient symptomatic relief on targeting Aβ. Many medications were introduced to target the inhibition of phosphorylation, glycosylation, or nitration of tau proteins to enhance their clearance, but the efficiency is still very low and many fail in phase I clinical trials [55]. Tau-targeted drugs are promising in the view of their expected AD disease-modifying effect; yet, are not achievable so far [3,55]. Being an essential glutamatergic cation channel gated receptor, NMDA mediated synaptic plasticity and transmission and acted in response to the Aβ binding to trigger neurotoxicity and neuronal death. Pallas-Bazarra et al. demonstrated that this action of NMDA was conducted via Tau protein, which transports tyrosine kinase Fyn to phosphorylate the GluN2B subunit of NMDA in synapses [55]. Drugs that combine both an action against tau proteins and Aβ are highly demanded, particularly, in the stage of preclinical AD when the neuronal changes are still reversible [56]. In the binding site of NMDA, six platycodin saponins revealed a better binding affinity than the controls, polygalacin D2, platycodin D, 3′-O-acetyl platyconic acid, platycodin D3, polygalacin D, and prosapogenin D with polygalacin D2 scoring the best Gibbs free energy of −7.755 Kcal/mol. It is worth mentioning that the same six molecules also superseded controls in the binding pockets of GSK-3β and BACE1.

A thorough conformational and docking analysis manifested the interaction of polygalacin D2 with the hydrophobic bed formed of leu280, leu377, met375, ile275, leu292, ile293, val355, and ala307, from which the last four amino acids exactly bonded to ifenprodil and ly-2811375 (Table 4). Polar interactions were displayed through 10 hydrogen bonds to residues Gln48, tyr351, asp353, lys296, gly354, gln291, and arg380, in which lys296 and gln291 also bonded to the controls. The hydroxyl groups in the tetrasaccharide side revealed contacts with gln48, tyr351, asp353, gly354, and asn294, while the aglycone core was located close to the nonpolar residues leu377 and met375. The diglucoside attached to position 3 formed two hydrogen bonds with lys296 and arg380, while gln 291 was unique in conducting another hydrogen bond to the 3-OH group and the anomeric oxygen of its attached glucose moiety. In most of the successfully NMDA docked molecules with a better than ifenprodil docking score, we noted the following interactions: hydrogen bonds with lys296, tyr351, gln291, and gly354. This primarily agreed with the results of Vashisth et al. [57].

**Figure 16:**
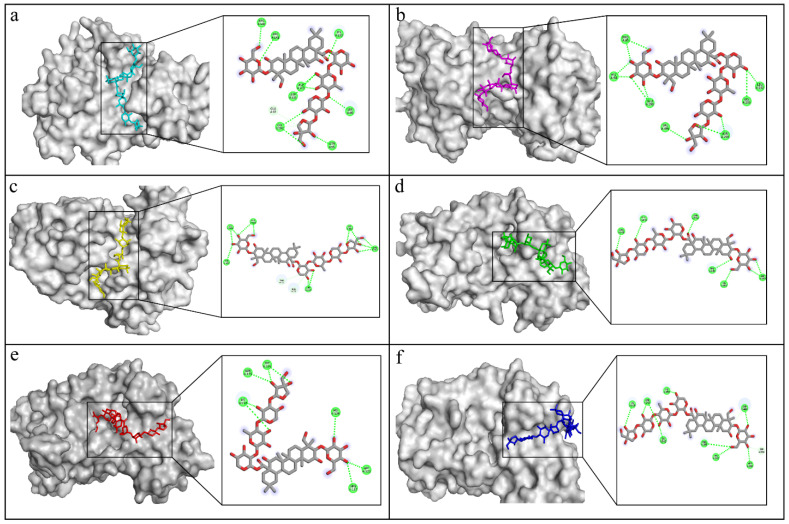
The molecular interactions of polygalacin D with protein targets. (**a**) GSK-3β, (**b**) NMDA receptor, (**c**) BACE1, (**d**) Synapsin I, (**e**) Synapsin II, (**f**) Synapsin III.

Another tau pathology targeting protein is the serine threonine kinase GSK-3β that is involved in the hyperphosphorylation of tau protein, the neurofibrillary tangles’ main component and hallmark of AD [58]. Additionally, it contributes to the control of amyloid-β (Aβ) peptide cell death and abnormal synaptic function [59]. Iwaloye et al. reported significant amino acids working for ATP-ligand recognition in the active site of GSK-3B as polar residues of LYS85, ASP200, and GLU51 as well as ASN186, ARG141, GLN185, LYS183, and ILE62. Asp200 was associated with the ATP phosphate interaction and was visible in most of the best-fitting platycodin saponins docked in the GSK-3B binding pocket [60]. Five docked saponins scored better binding free energy than the used controls of donepezil and ifenprodil; namely, platycodin D, polygalacin D, platycodin D3, platyconic acid B-lactone, and 3′-*O*-acetyl platyconic acid. Platycodin D attained a binding score of −7.147 Kcal/mol, which surpassed that of ifenoprodil and donepezil but not PF-04802, which revealed a ΔG value of −8.738 Kcal/mol. This was explained by the PF-04802 unique polar and nonpolar interactions to core residues in the GSK-3B pocket such as asp200, lys85, and val135 hydrogen bonds with distances of 2.64, 2.76, 2.29, and 2.41, respectively (Table 4). Halogen bonds were detected between chlorine and lys85 (3.75 A°) and asp200 (2.64 A°). Moreover, the hydrophobic bed in its vicinity comprised val70, phe67, cys199, ile62, leu188, ala83, leu132, val110, tyr134, val135, and tyr140 amino acids, among which ile62, leu188, cys199, and phe67 were clearly identified as powerful contacts in donepezil and ifenoprodil binding sites. Pi–cation interaction (4.39 A°) was noted between arg141 and the triazole ring. Furthermore, the orientation of platycodin D demonstrated that the aglycone part with the attached 3-glucose moiety was docked in the same side of the pocket as PF-04802; whereas, the trisaccharide attached unit was kept outside. The orientation of the two molecules despite varying sizes matched, where the aglycone part was closest to Arg141 in platycodin D instead of the triazole ring in PF-04802 with its Pi–cation interface. Furthermore, the chlorinated ring in PF-04802 occupied the same slot as the trisaccharide moiety in platycodin D but differed in forming two halogen carbon bonds and one hydrogen bond. In contrast, platycodin D trisaccharide extended five hydrogen bonds to core amino acids in the same slot, which justifies their binding energy values (Table 3). Polygalacin D assumed the same general orientation and interactions of platycodin D with the trisaccharide moiety extending outside the narrow groove and the 2-glucosyl aglycone impeded inside (Figure 16). However, arg148 showed interaction with the 6-hydroxy group of the glucose moiety, indicating a reversed aglycone positioning. This could contribute to the slight decrease in the ΔG value of polygalacin D and highlights the consequence of hydrophobic contacts in this enzymatic pocket.

## 4. Materials and Methods

### 4.1. Protein and Ligand Structures Retrieval

The Protein Data Bank was used for the retrieval of protein structures of synapsin I, synapsin II, synapsin III, BACE1, NMDA Receptor, and GSK-3β with PDB IDs 1PK8, 1I7L, 2P0A, 3K5F, 5EWJ, and 5K5N respectively. The missing residues and charges were detected and repaired by Modeller v9.22 [61]. PyMol 3.1 was used to visualize 3D structures of the proteins, ligands, and protein–ligand complexes. PubChem database [62] provided structural and functional aspects of compounds platycodin D, platycodin D3, 3″-O-acetylplatyconic acid A, prosapogenin D, and polygalacin D, polygalacin D2 with PubChem IDs 162859, 75251137, 101495503, 101596922, 96023791, and 70698218, respectively. The controls used with receptors GSK-3β, NMDA receptor, BACE1, synapsin I, synapsin II, and synapsin III were PF-04802367, ifenprodil, LY2811376, staurosporine, donzepil, and methylphenidate, respectively [63].

### 4.2. Ligand Preparation

The saponins compounds were retrieved from PubChem and processed using the LigPrep program in Schrödinger’s Maestro 2018 [64]. The OPLS_2005 force field was used to optimize the geometry of the ligands, ensuring they achieved energetically favorable conformations [21]. Energy minimization was applied to remove any unfavorable interactions or strained geometries.

### 4.3. Molecular Docking

The prepared saponins compounds were docked to BACE1 (PDB ID: 3K5F), GSK3β (PDB ID: 5K5N), NMDA receptor (PDB ID: 5EWJ), Synapsin I (PDB ID: 1PK8), Synapsin II (PDB ID: 1I7L), and Synapsin III (PDB ID: 2P0A). The crystal structures were prepared for docking using Protein Preparation Wizard of Maestro-2018 [65]. During receptor preparation, several stages were performed including the generation of disulfide bonds, the assignment of zero-order metal bonds, and the addition of hydrogens. The additional ligands and crystal water were also removed. In the optimization step, the pKa values of the ionizable group were optimized at pH 7.0 utilizing the PROPKA program in Schrödinger’s Maestro 2018 [62]. Finally, the OPLS_2005 forcefield was used for energy minimization. After the protein preparation, a three-dimensional grid was constructed at each receptor for site specific docking. The compounds were docked to the protein using the SP mode of glide [66].

### 4.4. MD Simulation

To analyze the protein ligand stability, an MD simulation of 100 ns was executed on the complexes of polygalacin D, polygalacin D2, and respective proteins. Solvation of the complexes was performed in a periodic box with a 10 Å size containing the TIP3P water molecules [67]. Counter ions of Na^+^ and Cl^−^ were introduced into the system to neutralize it. The minimization of the system was performed using the steepest decent method of 5000 steps following neutralization to remove steric conflicts. After minimization, the systems were prepared for the production run by equilibrating for 50,000 and 100,000 steps, respectively, at 310 K temperature at the NVT and NPT ensembles. The simulation was conducted with the Berendson thermostat and Parrinello-Rahman algorithms to maintain a constant temperature (310 K) and pressure (1 atm). By adjusting the time at τ P = 2.0 ps and τ T = 0.1 ps, the system was relaxed, and by applying the LINCS algorithm, the hydrogen atoms’ bond lengths were kept at their ideal lengths [68], whereas Verlet computed the non-bonded interactions [69]. To compute the electrostatic interactions beyond the short-range limit, the particle mesh Ewald approach was used [70]. In the x, y, and z dimensions, the periodic boundary conditions were imposed, and a production run was conducted on the system. Every 10 ps, the production run’s trajectory was saved and examined using the R BIO3D package 2.4-1.9000 and gromacs commands [71]. The CHARMM36 forcefield and the Gromacs simulation program 2024.2 were used to execute the simulation [72].

### 4.5. Quantum Chemical Investigation

We based our study primarily on the docking results, which revealed the interactions of the whole list of platycodin saponins to six key enzymes linked to AD pathology. In depth after-docking quantum chemical analysis was focused on chemical candidates with the highest binding free energy by conducting density functional theory (DFT) within Gaussian software 6.0 [73]. Geometry optimization was employed for the best two compounds, polygalacin D and polygalacin D2, by utilizing theory level B3LYP and the basis set of 6-31G, which allowed electronic energy calculations afterwards. Frontier molecular orbitals (FMOs) and molecular electrostatic potential (MEP) analysis were encompassed to showcase a thorough understanding of compounds reactivity in the biological system [74].

## 5. Conclusions

Currently, the scientific community is still searching for a cure for Alzheimer’s disease. With the current available computational advancements offering fast and cheaper approaches compared to the experimental one, platycodin saponins were investigated as promising drug candidates acting against neurodegenerative diseases, particularly, AD based on their previous bioactive profile and safety indexes. The studied compounds of polygalacin D and D2, and platycodin D, successfully inhibited AD enzymes like synapsins, NMDA, GSK-3B, and BACE1. Among the best results was polygalacin D2, which demonstrated a glide docking score of −7.755 Kcal/mol in the NMDA binding pocket verified through docking interactions and molecular dynamics simulation. Furthermore, the DFT quantum and electronic calculations were carried out for the three compounds to characterize their structures, which will assist their downstream development and formulation. In platycodin D, the moderate reactivity and electrophilicity was shown through its hydroxy ethyl group at carbon 1. The polygalacin D2 carbon2 diglucoside group was deemed responsible for its favored intermolecular interactions compared to polygalacin D. However, more in vitro studies, in vivo studies, and formulation studies are still needed to further study its effect, prove its therapeutic benefits, and improve its pharmacokinetics properties.

## Figures and Tables

**Table 1:** Selection of platycodon saponins in protein inhibitors using pharmacokinetic analysis.

No.	PubChem CID	Compounds	MW (g/mol)	HBD	HBA	logP	Drug-like
1	101596922	Prosapogenin D	603.817	6	8	3.62969	TRUE
2	162859	Platycodin D	1225.335	17	28	−5.3782	TRUE
3	385678065	Polygalacin D2	1371.477	19	32	−6.5264	FALSE
4	385678073	Polygalacin D3	1371.477	19	32	−6.5264	FALSE
5	46173919	Platycodin C	1267.372	16	29	−4.8074	TRUE
6	50900852	Platyconic acid B lactone	1383.444	18	33	−6.9818	FALSE
7	53317652	Platycodin D2	1387.476	20	33	−7.554	FALSE
8	70698202	Platycoside E	1549.617	23	38	−9.7298	FALSE
9	70698266	Deapioplatycodin D	1093.22	15	24	−3.8431	TRUE
10	70698289	Deapioplatycoside E	1417.502	21	34	−8.1947	FALSE
11	75251137	Platycodin D3	1387.476	20	33	−7.554	FALSE
12	96023791	Polygalacin D	1209.336	16	27	−4.3506	TRUE
13	70698190	Deapi-platycodin D3	1255.361	18	29	−6.0189	TRUE
14	46173910	Platycodin A	1267.4 g	16	29	−3.1	TRUE

**Table 2:** Docking interactions and binding free energies of platycodin saponins in the investigated enzymes.

Synapsin I	Binding Free Energy (Kcal/mol)	H-Bonds	Hydrophobic and VDW
polygalacin D2	−6.742	leu394, Arg186, Asp120, Glu121, Asn 334, Trp335, Asp283, Lys281 **(10)**	ile395, Pro393, Trp126, Met277, Gly276, Met297, Ser275. His123, Thr124, Asn338
3′-O-acetyl platyconic acid	−6.361	Asp309, Lys281, Gly333, Asn334, Asn 338, Thr339 **(8)**	Phe307, Pro265, Lys378, Trp335, Lys336, Gly340, Met277, Gly276, Ser275
LY281137	−6.916	Trp335	Arg328, Lys336, Thr337, Asn 338, Gly276, Ile385, Glu386, Lys279, Lys269, Leu375, Val267, Phe307, Ile308, Ala310, Asp313
PF-0480	−6.703	Lys269, Glu373, Ile308	Ala310, Leu375, Trp335, Asp309, Asn338, Phe307, Gly276
donepezil	−5.951	Lys279, Gly276, Trp335	Val388, Ile385, Leu375, Ala310, Ile308, Phe307, Pro306, Ile247, Val267
ifenoprodil	−4.672	Lys279	Trp335, Leu375, Ala310, Met392, Pro393, Val388, Ile385, Val267
**synapsin II**			
polygalacin D	−6.101	Lys337, trp336, asn335, glu122, asp121, arg187 **(7)**	ile396, leu395, pro304, ser392, met278, val281, lys282, gly279, met278, gly277, asn339, ser276
Polygalacin D2	−6.011	asp310(1.85, 2.50), lys379(2.05), lys282(1.89, 1.85, 2.08), asn339, ser276(1.88), glu197(1.65, 1.72) **(11)**	phe308, ala311, gly314, trp336, val281, met278, gly341, gly277, ala194, lys337, lys280, gly277, gly341
PF-0480	−6.839	ile309, phe386	val 376, trp 336, pro307, phe308, ile248, ala311, val268, lys379, lys374, lys337, lys280, ser276, asn335, glu306, asp310, asp314, ser331, arg329, lys270
LY-2811376	−5.727		phe386, val268, ile248, pro307, phe308, ile309, ala311, val376, trp336, lys280, lys270, lys337, lys374, arg329, gly277, ser276, asn339, thr338, glu306, asp314
ifenoprodil	−5.187	lys374, glu306, phe386, asp314	ile248, val376, pro307, phe308, ile309, val268, phe386, trp336, met389, pro394, lys270, lys337, arg329, arg316, thr338, asn339, ser276, gly277
donepezil	−4.879	lys374, glu306, phe386, asp34	ile248, val376, pro307, phe308, ile309, val268, phe386, trp336, met389
**synapsin III**			
polygalacin D	−6.145	ala254, asn313, gly312, lys260, phe286, asp288, asp358 **(8)**	met256, trp314, pro244, ala285, ile287, lys357, ser356
polygalacin D2	−5.882	ala254, asn313, lys260, asp288, asp358 **(8)**	met256, trp314, gly312, pro244,lys260, phe286, ile287, ser356, lys357, arg360, phe243
pf-0480	−6.361	lys248, ala254, lys352, trp314, ile287	met367, ile364, ala252, pro372, ala310, val354, phe286, ala285, val226, val246
ifenoprodil	−5.345	trp314	ala252, met367, ala254, ile364, val354, pro372, ala316, val246, ala285, phe286, ile287, val354
donepezil	−3.513	lys352, ser289	ala254, phe286, ile287, tyr291, ala316, trp314, val354, tyr291, val246, ile364
**GSK-3B**			
platycodin D	−7.147	asp200, asp181, lys183, ser66, asn186 **(6)**	val70, phe67, cys199, ile62, leu188, ala83, leu132, val110, tyr134, val135, tyr140
polygalacin D	−6.873	arg148, lys183, ile62, asp200, gly202, asn95 **(9)**	Tyr140, tyr222, ile217, val87, lys85, arg96, ser203, phe67, ser66, gly 65, gly63
platycodin D3	−6.514	ser66, asn64, gln185, arg141, thr138, pro136, tyr134 **(7)**	ile217, cys218, phe67, leu188, tyr140, pro136, val135, tyr134, ser219, asp181, lys183, phe67, gly65, gly62, ile62, lys60, gln72
platyconic acid B-lactone	−6.418	arg148, arg144, arg141, tyr140, glu137, tyr221, ser203, asp200, asp181, lys183 **(13)**	arg223, tyr222, tyr221, arg220, ser219, cys218, ser66, phe67, gly202, lys85, glu185, asn186
	−6.297	asp200, lys183, thr138, arg141, ser66, lys60, gln72 **(9)**	asp181, gln185, asn186, tyr140, phe67, gly65, asn64, gly63, ile62, val61, arg 148
PF-04820	−8.373	lys85, asp200, val135, arg141	phe67, val70, ala83, cys199, leu132, leu188, val110, tyr134, pro126, ile62
ifenprodil	−5.956	asn186	cys199, val110, ala83, leu132, ile62, tyr134, val135, pro134, phe67, val70, leu188
donepezil	−5.918	lys85	phe67, val70, ile62, phe201, cys199, met101, ala83, leu130, val110, leu132, tyr134, leu188, val135, pro136
**BACE1**			
polygalacin D2	−7.298	phe109, lys107, asn111, arg307, lys321, gly264, glu265, gln73, pro70, ile126, ser36, tyr198 **(15)**	phe47, phe108, ile110, leu263, tyr71, val69, ala127, gly34
polygalacin D	−5.607	Arg307, lys321, arg235, asp223, lys224 **(9 bonds)**	val309, tyr71, pro70, tyr198, Gly11, Gln12, Ser10, Lys107, Glu265, Thr72, Gln73, Ser327, Ser328, Thr329
3′-O-acetyl platyconic acid	−7.716	tyr198, tyr71, lys75, asn233, thr232, thr 231, gly230 **(7)**	trp197, lys224, arg128, pro70, thr72, gln73, gly74, thr329, ser328, gln326, er325, arg236, leu263, gly264, leu30, ile110, arg307, gln12
platyconic acid -B lactone	−6.93	lys75, tyr71, thr72, gln326, glu265, gly264, lys321, arg307, asn111 **(9)**	gly74, pro70, leu263, val309, phe47, ile110, phe109, val309, thr329, ser328, ser327, ser325, arg235, asn233, ser10, gly11, gln12, lys107
platycodin D3	−6.891	asp106, lys107, lys75, gly264, gln73, gly11, thr232, gly230 **(10)**	phe108, ile110, leu263, gly74, arg307, lys321, ser325, glu265, arg235, asn233, thr231, gln12, gly13, leu30, trp115
platycodin D	−6.758	gly11, thr232, the231, thr72, asp106 **(5)**	pro46, phe47, lys107, phe108, phe109, ile110, asn111, ile118, val332, ile226, tyr198, tyr71, gln73,gln12, arg235, lys321, arg307, glu265
ifenoprodil	−5.726	pro70, trp115, asp228	tyr71, val69, ile118, phe108, ile110, tyr198, ile226, leu30, val332
PF-04820	−5.147	tyr198, tyr71, asp228,	ile126, val332, phe108, ile110, trp115, ile118, pro70, val69, ile226, leu30
donepezil	−5.052	thr72	ile126, tyr198, ile226, val332, leu30, pro70, tyr71, ile118, phe108, ile110, trp115, arg128, gly34, ser35, asp228, asp32
LY-2811376	−5.018	gly11, thr72	trp115, ile118, leu30, tyr198, tyr71, phe108, ile110, asp228, ser35, asp32
**NMDA**			
polygalacin D2	−7.755	Gln48, tyr351, asp353, lys296, gly354, gln291, arg380 **(10)**	leu280, leu377, met375, ile275, leu292, ile293, val355, ala307
platycodin D	−7.296	leu382, gln371, gly354, asp353, tyr351, ser303 **(7)**	val383, ile293, Met375, val355, ala307, gln384, gln291, ser373, asn297, thr356, arg52, asp304
3′-O-acetyl platyconic acid	−7.214	pro95, thr122, lys37,glu298, lys296, asn294 **(10)**	pro96, pro95, ile41, ile293, ser93, ser34, thr35, ile293, ser299, asn297, gln291
platycodin D3	−7.183	glu246, tyr144, lys296, leu292, ser373, gln384 **(10)**	ser245, arg273, ser276, gly277, met125, arg124, thr123, thr122, asn297, gly295, asn294, ile293, gln291
polygalacin D	−6.736	pro95, glu298, lys296, leu292, asn294, val355 **(8)**	pro96, ser93, thr 122, ser34, asn297, gly295, ile293, gln 291, glu272, gln384, ser373
ifenoprodil	−5.29	ser393, gln291 **(2)**	leu292, ile293
prosapogenin D	−4.415	lys296, gln291**(4)**	ser299, glu298, asn297, asn294, ile293, ile387, gln384, ser373, met375
donepezil	−4.354	lys37, ser299 **(3)**	pro95, pro96, ile41, gly295
ly-2811375	−4.269	lys37, ser303 **(2)**	val355, ala45, ile41
pf-04820	−4.193	lys296 **(2)**	ile41, pro95, pro96

Green colour represents a heatmap that indicates the strength of bindings. Underline indicates matching between the native ligands and the docked compounds.

**Table 3:** Density functional theory calculations for selected compounds.

Compound	Polygalacin D	Prosapogenin D	Platycodin D	Polygalacin D2
**E_LUMO_ (eV)**	1.1064155	−0.02387	−0.026395057	0.122995523
**E_HOMO_ (eV)**	−6.7048887	−0.21197	−6.11385713	−6.03249504
**ΔE**	5.5984732	0.18826	6.087462073	5.909499517
**χ**	3.9056521	0.23538	3.070126094	2.954749759
**η**	3.9056521	−0.09405	3.043731037	3.077745282
**σ**	1.716714271	−2.25188569	2.008671941	0.324913178
**ω**	7.840523073	−124.3888	8.516100697	10.58547613
**dipole moment (DEBYE)**	7.8259	4.8371	7.2001	8.0721
**chemical potential (µ)**	2.7992366	0.11792	3.070126094	2.954749759
**electronic energy**	−17,794.64455	−7977.932898	−22,130.38172	−25,782.40283

**Table 4:** The glide scores of the saponin compounds against protein targets.

Compounds	GSK-3β	NMDA Receptor	BACE1	Synapsin I	Synapsin II	Synapsin III
Platycodin A	0	0	0	0	0	0
Platycodin C	0	0	0	0	0	0
Platycodin D	−7.147	−7.296	−6.758	−4.898	−3.712	−3.202
Deapioplatycodin D	0	0	0	0	0	0
Platycodin D2	0	0	0	0	0	0
Platycodin D3	−6.514	−7.183	−6.891	−5.995	−5.513	0
Deapioplatycodin D3	0	0	0	0	0	0
Deapioplatycoside E	0	0	0	0	0	0
Platyconic acid B lactone	−6.418	−6.623	−6.93	−6.058	−5.973	−4.801
3″-O-acetylplatyconic acid A	−6.297	−7.214	−7.716	−6.361	−6.078	−6.018
Prosapogenin D	−5.456	−4.415	−4.407	0	0	0
Polygalacin D	−6.873	−6.736	−5.607	−4.841	−6.101	−6.145
Polygalacin D3	0	0	0	0	0	0
Platycoside E	0	0	0	0	0	0
Polygalacin D2	−5.532	−7.755	−7.298	−6.742	−6.011	−5.882
Dimethyl 2-O-methyl-3-O-a-D-glucopyranosyl platycogenate A	−4.79	−3.918	−4.282	0	0	0
Platycodin V	0	0	0	0	0	0
PF-04802367	−8.373	/	/	/	/	/
Ifenprodil	/	−5.29	/	/	/	/
LY2811376	/	/	−5.018	/	/	/
Staurosporine	/	/	/	−3.52	/	/
Donzepil	/	/	/	/	−4.879	/
Methylphenidate (MPH)	/	/	/	/	/	−3.942

## Data Availability

Data will be available upon request.

## References

[1] Elhawary E.A., Moussa A.Y., Singab A.N.B. (2024). Genus Curcuma: Chemical and ethnopharmacological role in aging process. BMC Complement. Med. Ther..

[2] Better M.A. (2023). 2023 Alzheimer’s disease facts and figures. Alzheimers Dement..

[3] Limantoro J., de Liyis B.G., Sutedja J.C. (2023). Akt signaling pathway: A potential therapy for Alzheimer’s disease through glycogen synthase kinase 3 beta inhibition. Egypt. J. Neurol. Psychiatry Neurosurg..

[4] Abeysinghe A.A.D.T., Deshapriya R.D.U.S., Udawatte C. (2020). Alzheimer’s disease; a review of the pathophysiological basis and therapeutic interventions. Life Sci..

[5] Hampel H., Mesulam M.M., Cuello A.C., Farlow M.R., Giacobini E., Grossberg G.T., Khachaturian A.S., Vergallo A., Cavedo E., Snyder P.J. (2018). The cholinergic system in the pathophysiology and treatment of Alzheimer’s disease. Brain.

[6] Liu J., Chang L., Song Y., Li H., Wu Y. (2019). The role of NMDA receptors in Alzheimer’s disease. Front. Neurosci..

[7] Dou K.X., Tan M.S., Tan C.C., Cao X.-P., Hou X.-H., Guo Q.-H., Tan L., Mok V., Yu J.-T. (2018). Comparative safety and effectiveness of cholinesterase inhibitors and memantine for Alzheimer’s disease: A network meta-analysis of 41 randomized controlled trials. Alzheimers Res. Ther..

[8] Iqbal D., Alsaweed M., Jamal Q.M.S., Asad M.R., Rizvi S.M.D., Rizvi M.R., Albadrani H.M., Hamed M., Jahan S., Alyenbaawi H. (2023). Pharmacophore-based screening, molecular docking, and dynamic simulation of fungal metabolites as inhibitors of multi-targets in neurodegenerative disorders. Biomolecules.

[9] Deng M., Yan W., Gu Z., Li Y., Chen L., He B. (2023). Anti-neuroinflammatory potential of natural products in the treatment of Alzheimer’s disease. Molecules.

[10] Back M.K., Ruggieri S., Jacobi E., von Engelhardt J. (2021). Amyloid beta-mediated changes in synaptic function and spine number of neocortical neurons depend on NMDA receptors. Int. J. Mol. Sci..

[11] Ugale V.G., Bari S.B. (2016). Identification of potential Gly/NMDA receptor antagonists by cheminformatics approach: A combination of pharmacophore modelling, virtual screening and molecular docking studies. SAR QSAR Environ. Res..

[12] Clyde A. (2022). Ultrahigh throughput protein-ligand docking with deep learning. Methods Mol. Biol..

[13] Moussa A.Y., Alanzi A.R., Riaz M., Fayez S. (2024). Could mushrooms’ secondary metabolites ameliorate Alzheimer disease? A computational flexible docking investigation. J. Med. Food.

[14] Moussa A.Y., Alanzi A., Luo J., Chung S.K., Xu B. (2024). Potential anti-obesity effect of saponin metabolites from adzuki beans: A computational approach. Food Sci. Nutr..

[15] Moussa A.Y., Labib R.M., Ayoub N.A. (2013). Isolation of chemical constituents and protective effect of *Pistacia khinjuk* against CCl_4_–induced damage on HepG2 cells. Phytopharmacology.

[16] Torky Z.A., Moussa A.Y., Abdelghffar E.A., Abdel-Hameed U.K., Eldahshan O.A. (2021). Chemical profiling, antiviral and antiproliferative activities of the essential oil of *Phlomis aurea Decne* grown in Egypt. Food Funct..

[17] Sun A., Xu X., Lin J., Cui X., Xu R. (2015). Neuroprotection by saponins. Phytother. Res..

[18] Yang L., Hao J., Zhang J., Xia W., Dong X., Hu X., Kong F., Cui X. (2009). Ginsenoside Rg3 promotes beta-amyloid peptide degradation by enhancing gene expression of neprilysin. J. Pharm. Pharmacol..

[19] Yang Y., Chen W., Lin Z., Wu Y., Li Y., Xia X. (2024). Panax notoginseng saponins prevent dementia and oxidative stress in brains of SAMP8 mice by enhancing mitophagy. BMC Complement. Med. Ther..

[20] Xu T.Z., Shen X.Y., Sun L.L., Chen Y., Zhang B., Huang D., Li W. (2019). Ginsenoside Rg1 protects against H_2_O_2_-induced neuronal damage due to inhibition of the NLRP1 inflammasome signalling pathway in hippocampal neurons in vitro. Int. J. Mol. Med..

[21] Shivakumar D., Harder E., Damm W., Friesner R.A., Sherman W. (2012). Improving the prediction of absolute solvation free energies using the next generation OPLS force field. J. Chem. Theory Comput..

[22] Shen L.H., Zhang J.T. (2004). Ginsenoside Rg1 promotes proliferation of hippocampal progenitor cells. Neurol. Res..

[23] Zhao H.F., Li Q., Li Y. (2011). Long-term ginsenoside administration prevents memory loss in aged female C57BL/6J mice by modulating the redox status and up-regulating the plasticity-related proteins in hippocampus. Neuroscience.

[24] Zhu Y., Liang J., Gao C., Wang A., Xia J., Hong C., Zhong Z., Zuo Z., Kim J., Ren H. (2021). Multifunctional ginsenoside Rg3-based liposomes for glioma targeting therapy. J. Control. Release.

[25] Mao Y., Yuan W., Gai J., Zhang Y., Wu S., Xu E.-Y., Wang L., Zhang X., Guan J., Mao S. (2024). Enhanced brain distribution of Ginsenoside F1 via intranasal administration in combination with absorption enhancers. Int. J. Pharm..

[26] Wang C., Schuller Levis G.B., Lee E.B., Levis W.R., Lee D.W., Kim B.S., Park S.Y., Park E. (2004). Platycodin D and D3 isolated from the root of *Platycodon grandiflorum* modulate the production of nitric oxide and secretion of TNF-alpha in activated RAW 264.7 cells. Int. Immunopharmacol..

[27] Kiranmayee M., Rajesh N., Vidya Vani M., Khadri H., Mohammed A., Chinni S.V., Ramachawolran G., Riazunnisa K., Moussa A.Y. (2023). Green synthesis of Piper nigrum copper-based nanoparticles: In silico study and ADMET analysis to assess their antioxidant, antibacterial, and cytotoxic effects. Front. Chem..

[28] Kaya S., Putz M.V. (2022). Atoms-in-molecules’ faces of chemical hardness by conceptual density functional theory. Molecules.

[29] Branches A.D.S., da Silva J.N., de Oliveira M.D.L., Bezerra D.P., Soares M.B.P., Costa E.V., Oliveira K.M.T. (2024). DFT calculations, molecular docking, binding free energy analysis and cytotoxicity assay of 7,7-dimethylaporphine alkaloids with methylenedioxy ring in positions 1 and 2. Comput. Theor. Chem..

[30] Yamari I., Abchir O., Nour H., Khedraoui M., Rossafi B., Errougui A., Talbi M., Samadi A., El Kouali M., Chtita S. (2024). Unveiling Moroccan nature’s Arsenal: A computational molecular docking, density functional theory, and molecular dynamics study of natural compounds against drug-resistant fungal infections. Pharmaceuticals.

[31] Yovanno R.A., Chou T.H., Brantley S.J., Furukawa H., Lau A.Y. (2022). Excitatory and inhibitory D-serine binding to the NMDA receptor. eLife.

[32] Mayer M.L. (2017). The challenge of interpreting glutamate-receptor ion-channel structures. Biophys. J..

[33] Takashima A. (2006). GSK-3 is essential in the pathogenesis of Alzheimer’s disease. J. Alzheimers Dis..

[34] Wang C., Cui Y., Xu T., Zhou Y., Yang R., Wang T. (2023). New insights into glycogen synthase kinase-3: A common target for neurodegenerative diseases. Biochem. Pharmacol..

[35] Casiraghi A., Longhena F., Faustini G., Ribaudo G., Suigo L., Camacho-Hernandez G.A., Bono F., Brembati V., Newman A.H., Gianoncelli A. (2022). Methylphenidate analogues as a new class of potential disease-modifying agents for Parkinson’s disease: Evidence from cell models and alpha-synuclein transgenic mice. Pharmaceutics.

[36] Zhan Q., Zhang F., Sun L., Wu Z., Chen W. (2012). Two new oleanane-type triterpenoids from *Platycodi Radix* and anti-proliferative activity in HSC-T6 cells. Molecules.

[37] Nan F., Nan W., Yu Z., Wang H., Cui X., Jiang S., Zhang X., Li J., Wang Z., Zhang S. (2023). Polygalacin D inhibits the growth of hepatocellular carcinoma cells through BNIP3L-mediated mitophagy and endogenous apoptosis pathways. Chin. J. Nat. Med..

[38] Seo Y.S., Kang O.H., Kong R., Zhou T., Kim S.-A., Ryu S., Kim H.-R., Kwon D.-Y. (2018). Polygalacin D induces apoptosis and cell cycle arrest via the PI3K/Akt pathway in non-small cell lung cancer. Oncol. Rep..

[39] Kim M., Hwang I.G., Kim S.B., Choi A.J. (2019). Chemical characterization of balloon flower (*Platycodon grandiflorum*) sprout extracts and their regulation of inflammatory activity in lipopolysaccharide-stimulated RAW 264.7 murine macrophage cells. Food Sci. Nutr..

[40] Maesako M., Zoltowska K.M., Berezovska O. (2019). Synapsin 1 promotes Aβ generation via BACE1 modulation. PLoS ONE.

[41] Yan R., Fan Q., Zhou J., Vassar R. (2016). Inhibiting BACE1 to reverse synaptic dysfunctions in Alzheimer’s disease. Neurosci. Biobehav. Rev..

[42] Egbertson M., McGaughey G.B., Pitzenberger S.M., Stauffer S.R., Coburn C.A., Stachel S.J., Yang W., Barrow J.C., Neilson L.A., McWherter M. (2015). Methyl-substitution of an iminohydantoin spiropiperidine β-secretase (BACE-1) inhibitor has a profound effect on its potency. Bioorg Med. Chem. Lett..

[43] Joseph O.A., Babatomiwa K., Niyi A., Olaposi O., Olumide I. (2019). molecular docking and 3D osar studies of C000000956 as a potent inhibitor of Bace-1. Drug Res..

[44] Han B., Luo J., Xu B. (2024). Revealing molecular mechanisms of the bioactive saponins from edible root of *Platycodon grandiflorum* in combating obesity. Plants.

[45] Ji M.Y., Bo A., Yang M., Xu J.-F., Jiang L.-L., Zhou B.-C., Li M.-H. (2020). The pharmacological effects and health benefits of *Platycodon grandifloras*—A medicine food homology species. Foods.

[46] Moon M.K., Ahn J.Y., Kim S., Ryu S.Y., Kim Y.S., Ha T.Y. (2010). Ethanol extract and saponin of *Platycodon grandiflorum* ameliorate scopolamine-induced amnesia in mice. J. Med. Food.

[47] Zhang J.T., Xie L.Y., Shen Q., Liu W., Li M.-H., Hu R.-Y., Hu J.-N., Wang Z., Chen C. (2023). Platycodin D stimulates AMPK activity to inhibit the neurodegeneration caused by reactive oxygen species-induced inflammation and apoptosis. J. Ethnopharmacol..

[48] Choi J.H., Yoo K.Y., Park O.K., Lee C.H., Won M.-H., Hwang I.K., Ryu S.Y., Kim Y.S., Yi J.-S., Bae Y.-S. (2009). Platycodin D and 2″-O-acetyl-polygalacin D2 isolated from *Platycodon grandiflorum* protect ischemia/reperfusion injury in the gerbil hippocampus. Brain Res..

[49] Choi Y., Kang S., Cha S.H., Kim H.-S., Song K., Lee Y.J., Kim K., Kim Y.S., Cho S., Park Y. (2018). Platycodon saponins from Platycodi Radix (*Platycodon grandiflorum*) for the green synthesis of gold and silver nanoparticles. Nanoscale Res. Lett..

[50] Ha Y.W., Kim Y.S. (2008). Preparative isolation of six platycosides from *Platycodi Radix* by high-speed counter-current chromatography with evaporative light scattering detection. Planta Med..

[51] Shin K.C., Oh D.K. (2023). Biotransformation of platycosides, saponins from balloon flower root, into bioactive deglycosylated platycosides. Antioxidants.

[52] Nyakudya E., Jeong J.H., Lee N.K., Jeong Y.S. (2014). Platycosides from the roots of *Platycodon grandiflorum* and their health benefits. Prev. Nutr. Food Sci..

[53] Mirza F.J., Zahid S., Amber S., Sumera J.H., Asim N., Ali Shah S.A. (2022). Multitargeted molecular docking and dynamic simulation studies of bioactive compounds from *Rosmarinus officinalis* against Alzheimer’s disease. Molecules.

[54] Ji Y.J., Kang M.H., Kim G.S., Kim H.D., Jang G.Y. (2024). *Platycodon grandiflorum* exhibits anti-neuroinflammatory potential against beta-amyloid-induced toxicity in microglia cells. Front. Nutr..

[55] Pallas-Bazarra N., Draffin J., Cuadros R., Antonio Esteban J., Avila J. (2019). Tau is required for the function of extrasynaptic NMDA receptors. Sci. Rep..

[56] Pluta R., Ułamek-Kozioł M., Huang X. (2020). Tau protein-targeted therapies in Alzheimer’s disease: Current state and future perspectives. Alzheimer’s Disease: Drug Discovery.

[57] Vashisth M.K., Hu J., Liu M., Basha S.H., Yu C., Huang W. (2024). In-Silico discovery of 17alpha-hydroxywithanolide-D as potential neuroprotective allosteric modulator of NMDA receptor targeting Alzheimer’s disease. Sci. Rep..

[58] Dajani R., Fraser E., Roe S.M., Young N., Good V., Dale T.C., Pearl L.H. (2001). Crystal structure of glycogen synthase kinase 3 beta: Structural basis for phosphate-primed substrate specificity and autoinhibition. Cell.

[59] Sayas C.L., Ávila J. (2021). GSK-3 and tau: A key duet in Alzheimer’s disease. Cells.

[60] Iwaloye O., Elekofehinti O.O., Oluwarotimi E.A., Kikiowo B.I., Fadipe T.M. (2020). Insight into glycogen synthase kinase-3β inhibitory activity of phyto-constituents from *Melissa officinalis*: In silico studies. In Silico Pharmacol..

[61] Webb B., Sali A. (2021). Protein structure modeling with MODELLER. Methods Mol. Biol..

[62] Kim M.O., Nichols S.E., Wang Y., McCammon J.A. (2013). Effects of histidine protonation and rotameric states on virtual screening of M. tuberculosis RmlC. J. Comput. Aided Mol. Des..

[63] Alanzi A., Moussa A.Y., Mothana R.A., Abbas M., Ali I. (2024). In silico exploration of PD-L1 binding compounds: Structure-based virtual screening, molecular docking, and MD simulation. PLoS ONE.

[64] Yang Y., Yao K., Repasky M.P., Leswing K., Abel R., Shoichet B.K., Jerome S.V. (2021). Efficient exploration of chemical space with docking and deep learning. J. Chem. Theory Comput..

[65] Lu C., Wu C., Ghoreishi D., Chen W., Wang L., Damm W., Ross G.A., Dahlgren M.K., Russell E., Von Bargen C.D. (2021). OPLS4: Improving force field accuracy on challenging regimes of chemical space. J. Chem. Theory Comput..

[66] Friesner R.A., Banks J.L., Murphy R.B., Halgren T.A., Klicic J.J., Mainz D.T., Repasky M.P., Knoll E.H., Shelley M., Perry J.K. (2004). Glide: A new approach for rapid, accurate docking and scoring. 1. Method and assessment of docking accuracy. J. Med. Chem..

[67] Liu Y., Grimm M., Dai W.T., Hou M.C., Xiao Z.X., Cao Y. (2020). CB-Dock: A web server for cavity detection-guided protein-ligand blind docking. Acta Pharmacol. Sin..

[68] Qureshi K.A., Al Nasr I., Koko W.S., Khan T.A., Fatmi M.Q., Imtiaz M., Khan R.A., Mohammed H.A., Jaremko M., Emwas A.-H. (2021). In vitro and in silico approaches for the antileishmanial activity evaluations of actinomycins isolated from novel *Streptomyces smyrnaeus* strain UKAQ_23. Antibiotics.

[69] Hess B., Bekker H., Berendsen H.J.C., Fraaije J.G.E.M. (1997). LINCS: A linear constraint solver for molecular simulations. J. Comput. Chem..

[70] Grubmüller H., Heller H., Windemuth A., Schulten K. (1991). Generalized verlet algorithm for efficient molecular dynamics simulations with long-range interactions. Mol. Simul..

[71] Kholmurodov K., Smith W., Yasuoka K., Darden T., Ebisuzaki T. (2000). A smooth-particle mesh Ewald method for DL_POLY molecular dynamics simulation package on the Fujitsu VPP700. J. Comput. Chem..

[72] Grant B.J., Skjaerven L., Yao X.Q. (2021). The Bio3D packages for structural bioinformatics. Protein Sci..

[73] Huang J., MacKerell A.D. (2013). CHARMM36 all-atom additive protein force field: Validation based on comparison to NMR data. J. Comput. Chem..

[74] Frisch R., Gary M.J., Trucks G., Trucks S.H.B. (2016). Gaussian 6.0.

